# Associations of socioeconomic factors with parents’ awareness and acceptability of HPV vaccination in sub-Saharan Africa - a systematic review and meta-analysis

**DOI:** 10.1186/s12889-025-24550-x

**Published:** 2025-10-17

**Authors:** Alina John, Achenyo Peace Abbah, Ingvild Fossgard Sandøy

**Affiliations:** Centre for International Health, Department of Global Public Health and Primary Care, University of Bergen, Bergen, Norway

**Keywords:** Human papillomavirus, HPV vaccine, Cervical cancer, sub-Saharan Africa, Parents, Awareness, Acceptability, Systematic review, Meta-analysis, Socioeconomic status

## Abstract

**Background:**

Despite the effectiveness of Human Papillomavirus (HPV) vaccines in preventing cervical cancer, the low coverage of the vaccine remains a significant challenge, particularly in Sub-Saharan Africa, where the disease burden is high, and access to preventive services is limited. Socioeconomic determinants play a central role in shaping health behaviors and health outcomes. The uptake of the HPV vaccine is partly determined by parental decision-making, and this review aimed to examine whether socioeconomic factors are associated with awareness and acceptability of HPV vaccination among parents in sub-Saharan Africa.

**Methods:**

A systematic literature review was carried out according to the Preferred Reporting Items for Systemic Reviews and Meta-analysis guidelines. The databases searched included Medline, Embase, Web of Science, and the Cochrane Library. Three independent reviewers conducted the screening, study selection, data extraction and risk of bias assessment. Meta-analyses were performed and the certainty of evidence was evaluated using the Grading of Recommendations Assessment, Development and Evaluation.

**Results:**

The review included 26 articles, comprising 24 cross-sectional and two cohort studies. Seven studies examined parental awareness, 14 studies examined parental acceptability, and four studies examined both outcomes. Parents’ awareness of the HPV vaccine was associated with higher levels of education (OR = 3.81, 95% CI: 2.11–6.88, I^2^ = 84%, four studies), higher income (OR = 1.96, 95% CI: 0.72–5.29, I^2^ = 89%, four studies), moderate income (OR = 1.22, 95% CI: 0.67–2.23, I^2^ = 74%, four studies). Parents’ acceptance of HPV vaccination was associated with higher levels of education (OR = 2.06, 95% CI: 1.35–3.15, I^2^ = 77%, seven studies), higher income (OR = 2.16, 95% CI: 1.21–3.84, I^2^ = 77%, five studies), moderate income (OR = 1.33, 95% CI: 0.86–2.07, I^2^ = 74%, five studies) and urban residence (OR = 1.28, 95% CI: 0.84–1.96, I^2^ = 64%, five studies). The certainty of evidence ranged from low to very low.

**Conclusions:**

There may be a positive association between socioeconomic status, especially higher education and higher income, and parental awareness and acceptance of HPV vaccination. However the evidence is currently limited and inconsistent.

**Supplementary Information:**

The online version contains supplementary material available at 10.1186/s12889-025-24550-x.

## Introduction

Human papillomavirus (HPV) is a sexually transmitted infection (STI) that can cause a variety of diseases, ranging from genital warts to various cancers [1]. Approximately 630,000 new cervical cancer cases each year are linked to HPV, accounting for 4.5% of all cancer cases worldwide [2]. HPV is the leading risk factor for cervical cancer. Additional risk factors for cervical cancer include age, smoking and socioeconomic factors [3]. Furthermore, a weakened immune system increases the susceptibility to HPV infections and thus increases the risk of developing cervical cancer, particularly in individuals with HIV [4]. The World Health Organization (WHO) states that cervical cancer is the fourth most common cancer among women worldwide [5]. In 2020, there were an estimated 604,000 new cases of cervical cancer worldwide, with the majority of cases occurring in low- and middle-income countries (LMICs) [6].

Globally, the prevalence of HPV is highest in Africa (22%) and Central America (20%) followed by North America (11%), Europe (8%) and Asia (8%) [7, 8]. While HPV infections are most prevalent in LMICs among women under the age of 25 years, a rise in infections has been observed among women above the age of 45 in Africa, Central America and South America [9].

WHO estimates that 310,000 women died from cervical cancer in 2018 and that this number will reach 460,000 by 2040 [10]. Most of the increase in cervical cancer deaths is expected to occur in LMICs, primarily due to aging populations and population growth [11]. In sub-Saharan Africa (SSA), cervical cancer is one of the leading causes of death among women. Approximately 100,000 women are diagnosed with cervical cancer annually in SSA, and among these, 62% are expected to die due to the absence of routine screening and a lack of treatment [12].

In 2006, the Food and Drug Administration (FDA) approved the first HPV vaccine, and over the past two decades there have been significant advances in the global vaccine coverage. The WHO primarily recommends the HPV vaccines to young girls between the ages of 9–14 years [5] since the vaccine is most effective when given before being exposed to HPV. In 2007, Australia became the first country to introduce the HPV vaccine into its national immunization program. Since 2012, Gavi has played a key role in improving access to HPV vaccination for girls aged 9–14 in low-income countries [13]. By 2019, the HPV vaccine had been introduced in more than 100 countries [14]. Gavi reports that among the 48 SSA countries, only 19 have implemented HPV vaccination programs, while an additional 10 are in the planning stages [15]. In 2023, Nigeria launched a national HPV vaccination campaign that reached 12.3 million girls aged 9–14 over a period of nine months, marking one of the largest such initiatives in the region [16].

The coverage of the HPV vaccine and the success of HPV vaccination programs in SSA countries that have introduced HPV vaccination have unfortunately been limited due to the presence of various barriers. The limited availability and distribution of the HPV vaccine are one of the major obstacles. In 2022, WHO revised its recommendations based on emerging evidence that a single dose offers comparable protection to the traditional two-dose schedule [17]. However, in several countries in SSA, the shortage of vaccines has resulted in children remaining unvaccinated [18–20]. In addition, as school-based vaccination programs prioritize girls attending school, out-of-school girls face particular challenges in accessing immunization services [21]. This is exacerbated by challenges faced by the health sector in this region [22], leading to inadequate provision of health services such as school-based immunization [23].

Lack of awareness and knowledge regarding the risks associated with cervical cancer and the effectiveness of the HPV vaccine for cancer prevention is another major barrier, according to a systematic review [24]. As parental consent is generally required for the vaccination of children, parents’ perceptions of the vaccines are likely to influence vaccination coverage. If parents have low levels of knowledge about the vaccine’s benefits, this may lead to misconceptions or concerns and is likely to affect parents´ acceptance of the HPV vaccine [25]. In Kenya, a cross-sectional survey found that mothers’ fear of promoting early sexual activity or risky sexual behavior discouraged them from allowing their daughters to be vaccinated against HPV [26]. Similarly, a study conducted in Tanzania found that misconceptions regarding the risk of infertility reduced the acceptability of the vaccine among parents [27].

Previous studies indicate that individuals from disadvantaged backgrounds may be at a higher risk of contracting HIV and STIs than those from higher socioeconomic groups [28–30]. A few studies suggest that the same may be the case for HPV infection [31]. It is believed that socioeconomic disparities may also influence parents’ awareness and acceptance of the HPV vaccine for their daughters. Studies have indicated that higher socio-economic status may be linked with lower vaccine hesitancy among parents [32] and greater access to information [33] thereby potentially leading to higher awareness and acceptability of the HPV vaccine for their daughters. Research conducted in Uganda found that girls from higher socio-economic backgrounds, such as higher wealth index [34] and higher educational level among mothers, exhibit higher rates of HPV vaccination [35]. By conducting a systematic review, insight can be gained into the potential relationship between parental socioeconomic status and their awareness and acceptance of the HPV vaccine. This will inform the design and implementation of effective vaccination programs in sub-Saharan Africa aimed at reducing disparities between different socioeconomic groups. This review aims to examine whether socioeconomic factors are associated with awareness and acceptability of HPV vaccination among parents in sub-Saharan Africa, and to explore whether the associations are different in the period before versus after the implementation of national HPV vaccination programs, as well as between mothers and fathers.

## Methods

This systematic review was conducted in accordance with the PRISMA 2020 guidelines (Preferred Reporting Items for Systematic Reviews and Meta-Analyses), provided in Supplementary file (*see Appendix M*) [36]. The protocol of this review was registered in PROSPERO the International Prospective Register of Systematic Reviews (ID: CRD42022366608).

### Eligibility criteria

The following inclusion criteria were applied:

- Context: All countries in sub-Saharan Africa (Angola, Benin, Botswana, Burkina Faso, Burundi, Cabo Verde, Cameroon, Central African Republic, Chad, Comoros, Democratic Republic of Congo, Republic of Côte d’Ivoire, Equatorial Guinea, Eritrea, Eswatini, Ethiopia, Gabon, Gambia, Ghana, Guinea, Guinea Bissau, Kenya, Lesotho, Liberia, Madagascar, Malawi, Mali, Mauritania, Mauritius, Mozambique, Namibia, Niger, Nigeria, Rwanda, Sao Tome and Principe, Senegal, Seychelles, Sierra Leone, Somalia, South Africa, South Sudan, Sudan, Swaziland, Tanzania, Togo, Uganda, Zambia, Zimbabwe).
- Participants: Parents, caregivers or legal guardians with daughters.
- Outcomes: Awareness of HPV vaccination and acceptability of HPV vaccination.
- Socioeconomic factors as independent variables: education, income, occupation, area of residence (urban/rural).
- Study design: cross-sectional studies, cohort studies.
- Language: Published in English.
- Publication year: 2000–2024.

### Information sources

Systematic searches were conducted in the databases Medline, Embase, Web of Science, and Cochrane Library databases up to 13th May 2024. The search strategy was developed based on the eligibility criteria using a combination of Medical Subject Headings (MeSH) and free-text terms related to HPV, HPV vaccination, socioeconomic factors, parents/guardians, and countries in SSA. To ensure a comprehensive search strategy, a qualified librarian was consulted to review the search terms, search syntax and search field tags. The librarian also provided guidance in creating tailored search strategies for each database. The individual search strategies for each database are provided in the supplementary file, *Appendix A*.

### Screening

After conducting a thorough search across four databases, the results were imported into EndNote, and duplicates were eliminated. The titles and abstracts were screened by three independent reviewers (AJ, APA and IFS) in Rayyan. The same three reviewers independently identified potentially relevant studies and assessed their eligibility in full text. Disagreements were solved through discussions.

### Data collection process

Data extraction was also carried out by the three reviewers (AJ, APA and IFS) using customized data extraction forms. The primary outcomes of interest were awareness and acceptability of HPV vaccination among parents in SSA.

Awareness was defined as having heard of the HPV vaccine or recognizing it as a preventive measure against HPV-related diseases, while acceptability was defined as the willingness of parents to vaccinate their daughters against HPV. Estimates of the association between the socioeconomic factors (education, income, occupation, and urban/rural area of residence) and the outcomes of interest were collected from bivariate and multivariable analyses from the included articles. Such data was extracted separately to capture any potential differences in reported information by mothers and fathers.

There were differences in how socioeconomic factors were categorized across the included studies. Certain categories were combined to ensure consistency and comparability when conducting the meta-analyses. For education, primary and secondary levels were combined and compared to the tertiary level. For studies with four or more income categories, we grouped the responses into low, moderate, and high income based on approximate percentiles within each study: low income was defined as below the 25th percentile, moderate between the 25th and 75th percentiles, and high above the 75th percentile. This approach allowed for comparability across studies with different income scales. No currency conversions were performed, as income was analyzed within-study. The lowest category was used as the reference group.

The measures of association used in the included studies were mainly crude odds ratios (OR) and adjusted odds ratios (AOR), along with their respective 95% confidence intervals (CI). For studies that reported chi-square values as a measure of association, we manually calculated odds ratios (ORs) to maintain consistency in the presentation of results.

### Risk of bias assessment

The risk of bias assessment was conducted by AJ and APA independently using the Joanna Briggs Institute (JBI) Critical Appraisal Tools for cross-sectional and cohort studies [37]. Any disagreements were resolved through discussion until a consensus was reached. This tool assessed multiple domains of study quality, including eligibility criteria, sample selection, measurement validity and reliability, confounding factors, follow up and statistical analysis to determine the risk of bias. The questions used to conduct the risk of bias assessment are provided in the supplementary file (*see Appendix B and C*).

The study’s risk of bias was assessed by categorizing responses to each question as Yes (Y), No (N), or Unclear (U). A scoring system was incorporated to summarize the assessment of the risk of bias more easily. The JBI checklist for cross-sectional studies consisted of 8 criteria, with 1 point assigned for each criterion fulfilled and 0.5 points if unclear. The total score for each study was classified into three quality categories based on the following criteria: 0-3.5 points were considered low quality, 4.0-6.5 moderate quality, and scores between 7.0 and 8.0 were regarded as high quality. The JBI checklist consisted of 11 criteria for cohort studies, with the same score assignment as for the cross-sectional studies. Studies with total scores between 0 and 4.5 points were considered low quality, between 5.0 and 8.5 points moderate quality, and scores between 9.0 and 11.0 points were considered high quality. The results of the risk of bias assessments were used to inform the interpretation of findings and contributed to the certainty grading using the Grading of Recommendations, Assessment, Development and Evaluation (GRADE).

### Synthesis methods

Articles included in the systematic review were eligible for meta-analysis if they reported comparable socioeconomic categories and provided sufficient quantitative data on the relevant outcomes to allow pooling. Studies without comparable categories or necessary data were included only in the narrative synthesis.

For income, two meta-analyses were conducted, which compared both moderate and high incomes to low income. The overall effect size for each meta-analysis was estimated by using the Mantel-Haenszel method using RevMan 5.4.

To account for potential heterogeneity, random-effects models were employed when there was high heterogeneity among the studies, while fixed-effects models were used when heterogeneity was low. Subgroup analyses were conducted to investigate potential sources of heterogeneity among the studies. These analyses were performed according to gender (mothers vs. fathers) or the timing of the study (before or after the introduction of national HPV vaccination programs) for meta-analyses that exhibited substantial or high heterogeneity.

In our meta-analyses, we used crude data extracted from the studies, manually entering sample sizes and event counts into RevMan. The software then calculated crude odds ratios (COR) using these data. Adjusted odds ratios (AOR) were inconsistently reported across studies and therefore were not directly used in the meta-analyses. This approach allowed for greater consistency and comparability between studies.

### Certainty assessment

The certainty of the evidence for each meta-analysis was assessed using GRADE. To assess for potential publication bias, funnel plots were generated using RevMan 5.4, provided in the supplementary file (*see Appendix K*). As the included studies were observational, the initial level of certainty was low. The reasons for downgrading were:

#### Risk of bias

Downgraded if the overall risk of bias was considered serious or if a study with a high risk of bias had a significant impact on the meta-analysis results.

#### Inconsistency

Downgraded if the heterogeneity was substantial to considerable (*I*^*2*^ = 50–100%) [38].

#### Imprecision

Downgraded if the total sample size was considered small or if the confidence interval was wide.

#### Indirectness

Downgraded if there were concerns about the generalizability or relevance of the findings to the population of interest.

#### Publication bias

Downgraded if publication bias was detected graphically by using funnel plots.

## Results

### Study selection

**Fig. 1:**
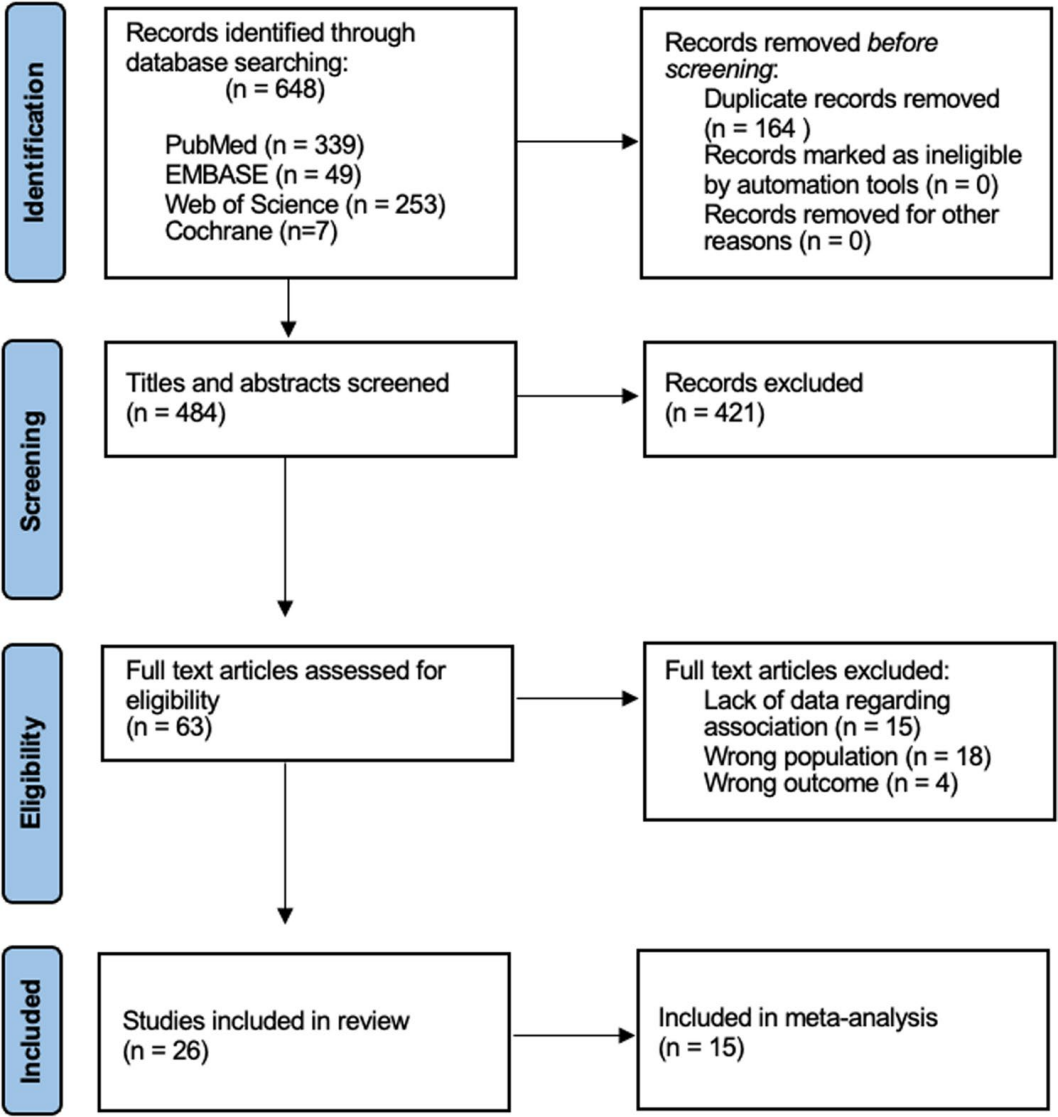
PRISMA 2020 flowchart displaying the selection process

A total of 648 studies were identified from the four databases, as presented in Fig. 1. Following the removal of 164 duplicates, the titles and abstracts of 484 studies were screened. A total of 63 potentially relevant studies were identified and assessed in full text. After the full-text assessment, 37 articles were excluded as they lacked data regarding the associations of interest or focused on the wrong population or outcomes. Ultimately, 26 studies were deemed eligible and included in this systematic review. 

### Study characteristics

An overview of the 26 included studies is provided in Table 1. The studies were primarily from Nigeria (*n* = 11), followed by Ethiopia (*n* = 8), Kenya (*n* = 2), Zimbabwe (*n* = 1), Uganda (*n* = 1), Botswana (*n* = 1), Zambia (*n* = 1) and South Africa (*n* = 1). Most of the studies were cross-sectional studies (*n* = 24), while the remaining were cohort studies (*n* = 2). For the outcomes, 14 solely examined acceptability, eight studies solely examined awareness, and four studies examined both. Across the included studies, 24 examined education, 13 examined income, 13 examined occupation, and seven examined place of residence. All but six studies examined more than one socioeconomic factor. 10 studies focused exclusively on mothers, while 16 included both parents. The mean age reported across studies was 34.22 years in studies including only mothers, and 35.91 years in studies including both parents. Sample sizes varied from 148 to 1002 participants. Ten studies were conducted before the introduction of national vaccination programs while nine studies were conducted after the introduction.

The quality of the included studies was assessed using the JBI checklist (see Supplementary file). Among the 24 cross-sectional studies, three were rated as high quality, 20 as moderate quality, and one as low quality (*see Table S1*,* Appendix D*). Both cohort studies were assessed as moderate quality (*see Table S2*,* Appendix E*).

**Table 1:** Characteristics of included studies

Authors, publication year	Location	Study design	Study year	Participants	Age (mean)	Sample size	Outcome	Socioeconomic factors addressed	Overall quality
Akinleye et al., 2020 [39]	Nigeria	Cross-sectional	2018	Parents of adolescent girls	43.9 ± 7.3	301	Acceptability	Education	Moderate
Alene et al., 2020 [40]	Ethiopia	Cross-sectional	2019	Parents of daughters aged 9–17 years	39.0 ± 0.30	899	Acceptability	Residence Education Income	High
Anyaka et al., 2024 [41]	Nigeria	Cross-sectional	2019	Parents with daughters aged 9–14 years	43.7 ± 9.43	509	Awareness Acceptability	Education	Moderate
Aragaw et al., 2023 [42]	Ethiopia	Cross-sectional	2021/2022	Parents with daughters aged 9–14 years	40.9 ± 6.72	721	Acceptability	Education Income Occupation	Moderate
Azuogu et al., 2019 [43]	Nigeria	Cross-sectional	2018	Mothers of adolescent daughters	42.0 ± 8.0	290	Awareness	Education	Moderate
Dairo et al., 2016 [44]	Nigeria	Cross-sectional	NA	Parents of adolescent girls	37.3 ± 6.5	612	Acceptability	Income Occupation	Moderate
Destaw et al., 2021 [45]	Ethiopia	Cross-sectional	2020–2021	Parents of daughters aged 9–13 years	28.6 ± 6.0	502	Acceptability	Residence Income Education	Moderate
Dereje et al., 2021 [46]	Ethiopia	Cross-sectional	2021	Parents or guardians of daughters aged 9–17 years	39.0 ± 9.9	430	Acceptability	Income	Moderate
DiAngi et al., 2011 [47]	Botswana	Cross-sectional	2009	Parents of adolescent daughters	NA	376	Acceptability	Income Education Residence	Moderate
Enebe et al., 2021 [48]	Nigeria	Cross-sectional	2017	Female secondary school teachers	37.46 ± 9.15	377	Awareness	Education Income	Moderate
Ezenwa et al., 2013 [49]	Nigeria	Cross-sectional	2012	Mothers of adolescent daughters	40.1 ± 6.9	290	Awareness	Education	Moderate
Humnesa et al., 2022 [50]	Ethiopia	Cross-sectional	2021	Parents with daughters aged 9–14 years	35.13 ± 7.69	619	Awareness	Education Income Residence	Moderate
Kolek et al., 2022 [51]	Kenya	Cross-sectional	2020	Parents of daughters aged 9–14 years	NA	195	Acceptability	Occupation Education	High
Lubeya et al., 2023 [52]	Zambia	Cross-sectional	2021	Parents of daughters aged 15–18 years	45.7 ± 1.4	400	Acceptability	Education Income Occupation	Moderate
Mihretie et al., 2022 [53]	Ethiopia	Cross-sectional	2020–2021	Parents of daughters aged 9–14 years	36.41 ± 5.8	638	Acceptability Awareness	Education Occupation	Moderate
Milondzo et al., 2021 [54]	South Africa	Cross-sectional	2018	Caregivers of girls aged > 9 years	NA	615	Acceptability	Education Occupation	Moderate
Morhason-Bello et al., 2015 [55]	Nigeria	Cross-sectional	2012	Women of reproductive age (18–49 years)	29.8 ± 8.2	1002	Awareness	Occupation Education Income	Moderate
Ndejjo et al., 2017 [56]	Uganda	Cross-sectional	NA	Mothers aged 25–49 years	32.9 ± 6.7	900	Acceptability	Residence Education Occupation Income	Moderate
Nkwonta et al., 2021 [57]	Nigeria	Pre-/posttest design	NA	Men and women aged 18–65 years	37.8 ± 11.1 36.1 ± 9.43	281	Awareness	Education Income	Moderate
Okunade et al., 2017 [58]	Nigeria	Cross-sectional	2015	Mothers aged 20–65 years	35.7 ± 9.74	148	Awareness Acceptability	Education	Moderate
Okunowo et al., 2021 [29]	Nigeria	Cross-sectional	2019	Mothers aged 21–65 years	41.9 ± 13.1	208	Acceptability	Education Occupation	Moderate
Rabiu et al., 2020 [30]	Nigeria	Cross-sectional	2013	Parents of adolescent girls	NA	318	Acceptability	Education Occupation	Low
Sinshaw et al., 2022 [59]	Ethiopia	Cross-sectional	2021	Parents of eligible girls	39.4 ± 8.95	601	Awareness Acceptability	Income Education Occupation	High
Tsige et al., 2024 [60]	Ethiopia	Cross-sectional	2023	Mothers with daughters aged 14–18 years	NA	607	Awareness	Education Occupation	Moderate
Vermandere et al., 2014 [61]	Kenya	Cohort	2013	Mothers of girls aged 9–14 years	NA	287	Acceptability	Education Residence	Moderate
Zibako et al., 2021 [62]	Zimbabwe	Cross-sectional	2019–2020	Mothers of girls aged 9–14 years	34 ± 5.18	406	Awareness	Occupation Residence Education	Moderate

### Association between socioeconomic factors and parents’ awareness of HPV vaccination

#### Association between education and awareness of HPV vaccination

Twelve studies examined the relationship between educational level and awareness of the HPV vaccine (Table 2). A study from Nigeria conducted in 2012 reported that more than half of the parents with tertiary education had heard of the HPV vaccine, while those with primary or no formal education had no knowledge of the vaccine [49]. Another study from Ethiopia conducted in 2021 reported that mothers with a degree and those with diplomas were about 8 and 4 times more likely to have good knowledge about the HPV vaccine, respectively, compared to mothers who could not read and write [59]. Four of the studies were included in the meta-analysis. Since estimates were available for both mothers and fathers in one of the studies, both were included in this analysis [59].

**Table 2:** Association between education and parents’ awareness of HPV vaccination

Author, publication year	Education	*N* (%)	OR [95% CI]	AOR [95% CI]
Azuogu et al., 2019 [43]	Primary education or less Secondary education or more	18 (22.8) 66 (61.1)	Ref 5.33 (2.77–10.23)	***
Anyaka et al., 2024 [41]	None Primary Secondary Tertiary	1 (1.4) 1(0.7) 2 (1.0) 4 (4.2)	Ref. 0.46 (0.03–7.52) 0.74 (0.07–8.25) 3.08 (0.34–28.14)	***
Enebe et al., 2021 [48]	Secondary Tertiary Post-graduate	2 (20.0) 14 (10.6) 5 (7.6)	Ref 0.47 (0.09–2.46) 0.33 (0.05–1.98)	***
Ezenwa et al., 2013 [49]	None Primary Secondary Tertiary	0 (0.0) 0 (0.0) 12 (8.9) 45 (44.1)	Ref 8.22 (4.04–16.72)	***
Humnesa et al., 2022 [50]	Illiterate Primary Secondary College and above	46 (32.9) 39 (37.5) 63 (35.2) 94 (48.0)	Ref 1.23 (0.72–2.08) 1.11 (0.69–1.77) 1.89 (1.2–2.96)	Ref 1.65 (0.80–3.41) 1.52 (0.71–3.41) 1.90 (0.67–5.39)
Mihretie et al., 2022 [53]	No formal education Primary education Secondary and above	72 (40.9) 77 (33.9) 75 (31.9)	Ref 0.74 (0.49–1.11) 0.67 (0.45–1.01)	Ref 0.76 (0.50–1.15) 0.70 (0.46–1.05)
Morhason-Bello et al., 2015 [55]	Secondary or less Tertiary	19 (2.9) 26 (8.6)	Ref 3.22 (1.75–5.91)	
Nkwonta et al., 2021 [57] (M)	No formal education Primary education Secondary education Tertiary education	*	**	0.25 (0.12–0.46) 0.15 (0.06–0.36) 0.53 (0.35–0.82) Ref
Nkwonta et al., 2021 [57] (F)	No formal education Primary education Secondary education Tertiary education	*	**	0.17 (0.09–0.30) 0.28 (0.19–0.35) 0.54 (0.40–0.61) Ref
Okunade et al., 2017 [58]	Less than tertiary At least tertiary	1 (9.1) 27 (84.4)	Ref 54 (5.6–520)	***
Sinshaw et al., 2022 [59] (M)	Cannot read and write Read and write only Primary school Secondary school Diploma Degree and above	13 (16.3) 17 (21.5) 29 (31.5) 53 (48.6) 96 (64.9) 78 (83.9)	Ref 1.41 (0.64–3.15) 2.37 (1.13–4.97) 4.88 (2.42–9.85) 9.52 (4.81–188) 26.8 (11.91–0.32)	Ref 0.86 (0.31–2.42) 1.40 (0.52–3.74) 2.47 (0.94–6.50) 3.54 (1.17–10.75) 7.69 (1.84–32.17)
Sinshaw et al., 2022 [59] (F)	Cannot read and write Read and write only Primary school Secondary school Diploma Degree and above	2 (16.7) 7 (18.4) 22 (31.4) 32 (37.2) 69 (55.6) 107 (72.8)	Ref 1.13 (0.20–6.34) 2.30 (0.46–11.35) 2.96 (0.61–14.38) 6.27 (1.32–29.82) 13.38 (2.81–63.72)	Ref 0.64 (0.09–4.70) 1.16 (0.17–7.73) 0.98 (0.15–6.23) 1.56 (0.24–10.22) 2.07 (0.29–14.66)
Tsige et al., 2024 [60]	Unable to read and write Read and write only Primary/secondary Diploma or above	*	Ref 3.27 (2.13–3.96) 3.87 (2.15–8.85) 21.80 (10.90–31.32)	Ref 2.39 (0.42–4.74) 3.47 (0.34–6.50) 9.21 (0.82–12.16)
Zibako et al., 2021 [62]	Primary Secondary Tertiary	*	Ref 4.20 (2.25–7.84) 7.75 (2.04–29.45)	***

*(M) Indicates mothers*,* (F) Indicates fathers*

* Information on N (%) was missing

** information on OR was missing

*** information on AOR was missing

**Fig. 2:**
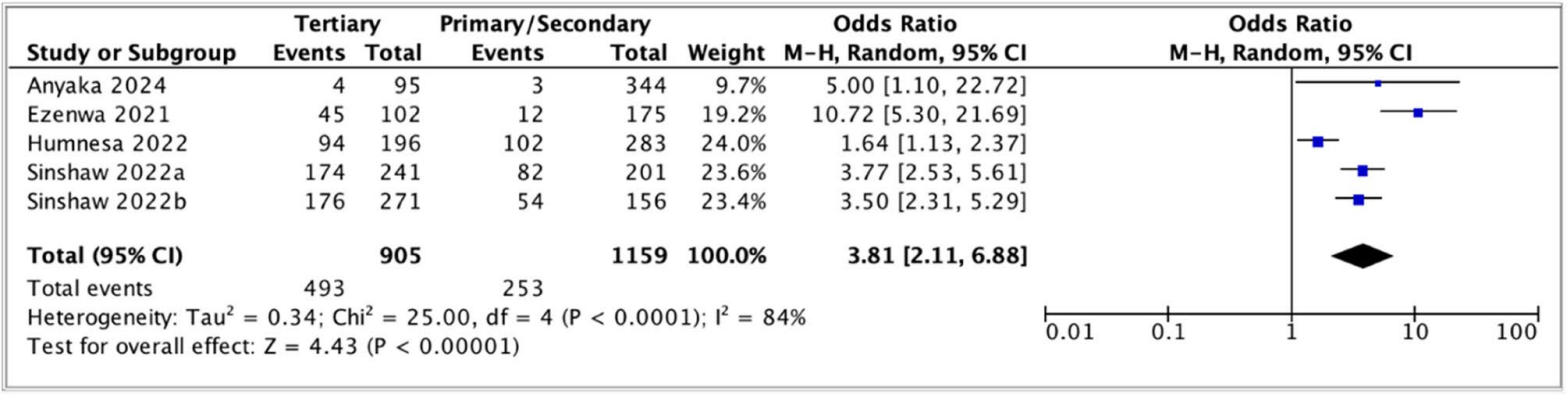
Meta-analysis of association between education and parental awareness of HPV vaccination

**Fig. 3:**
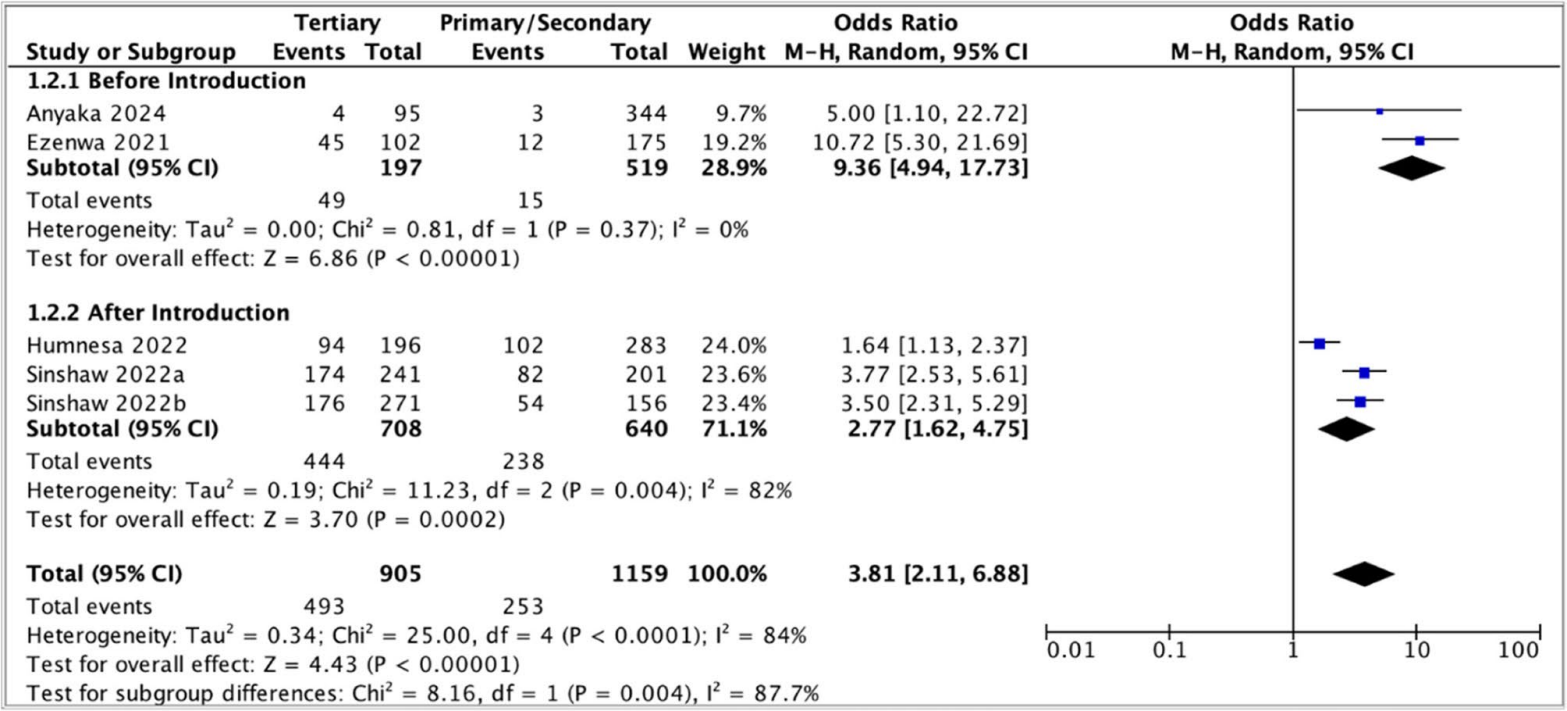
Subgroup analysis comparing association between aware of HPV vaccination and education, before and after the introduction of national HPV vaccination programs

The pooled odds ratio (3.81, 95% CI: 2.11–6.88) suggested that parents with tertiary education had higher awareness of HPV vaccination than those with lower educational levels (Fig. 2). However, high heterogeneity was observed (*I*^2^ = 84%). This heterogeneity was investigated by stratifying according to the timing of the study (before and after the introduction of a national HPV vaccination program). The pooled odds ratio was higher and the heterogeneity was low in the before-introduction subgroup (9.36, 95% CI: 4.94–17.73), but the confidence intervals were quite wide (Fig. 3).

#### Association between income and awareness

As presented in Table 3, five studies examined the association between monthly income and the level of parental awareness of HPV vaccines [48, 50, 55, 57, 59]. Four of these studies were included in the meta-analysis, and estimates from both parents were included from one study [59].

**Table 3:** Association between income and parents’ awareness of HPV vaccination

Author, publication year	Income	*N* (%)	OR [95% CI]	AOR [95% CI]
Enebe et al., 2021 [48]	< ₦18,000 ₦18 000–49 999 ₦50 000–99 999 ₦100 000–199 999 >₦199 999	1 (10.0) 14 (14.7) 2 (2.4) 2 (12.5) 2 (40.0)	Ref 1.56 (0.18–13.25) 0.23 (0.02–2.73) 1.29 (0.10–16.34) 6.00 (0.39–92.82)	***
Humnesa et al., 2022 [50]	Poor Medium Rich	85 (43.4) 74 (34.1) 83 (40.3)	Ref 0.68 (0.45–1.01) 0.88 (0.59–1.31)	***
Morhason-Bello et al., 2015 [55]	₦10 000 and less ₦11 000–20 000 >₦20 000 ₦ Undisclosed	8 (2.9) 10 (5.5) 9 (6.9) 19 (4.8)	Ref 1.97 (0.76–5.10) 2.49 (0.94–6.61) 1.71 (0.74–3.96)	***
Nkwonta et al., 2021 [57] (M)	₦50 000 ₦50 000–100 000 >₦100 000	*	**	Ref 0.75 (0.59–0.95) 1.40 (1.18–1.62)
Nkwonta et al., 2021 [57] (F)	₦50 000 ₦50 000–100 000 >₦100 000	*	**	Ref 0.74 (0.65–0.95) 1.62 (1.40–1.86)
Sinshaw et al., 2022 [59] (M)	< Br 600 Br 601–1650 Br 1651–3200 Br 3201–5250 Br 5251–7800 Br 7801–10 900 >Br 10 900	30 (31.9) 49 (34.0) 52 (33.3) 68 (74.7) 57 (77.0) 25 (69.4) 5 (83.3)	Ref 1.10 (0.63–1.92) 1.07 (0.62–1.84) 6.31 (3.32–11.98) 7.15 (3.57–14.32) 4.85 (2.11–11.13) 10.67 (1.19–95.35)	Ref 0.83 (0.39–1.74) 0.74 (0.32–1.69) 2.21 (0.64–7.61) 2.42 (0.59–9.92) 1.21 (0.24–6.16) 0.64 (0.45–9.05)
Sinshaw et al., 2022 [59] (F)	< Br 600 Br 601–1650 Br 1651–3200 Br 3201–5250 Br 5251–7800 Br 7801–10 900 >Br 10 900	1 (12.5) 8 (61.5) 31 (37) 58 (41.4) 60 (53.1) 72 (68.6) 9 (64.3)	Ref 11.2 (1.04–120) 4.1 (0.48–34.86) 4.95 (0.59–41.34) 7.93 (0.94–66.53) 15.3 (1.81–129) 12.6 (1.19–133)	Ref 9.4 (0.40–221) 2.92 (0.17–50.58) 2.31 (0.14–38.55) 1.23 (0.07–21.73) 1.57 (0.09–28.21) 1.09 (0.05–25.79)

*(M) indicates mothers*,* (F) indicates fathers.*

*₦* Nigerian Naira, *Br* Ethiopian Birr.

* Information on N (%) was missing

** information on OR was missing

*** information on AOR was missing

**Fig. 4:**
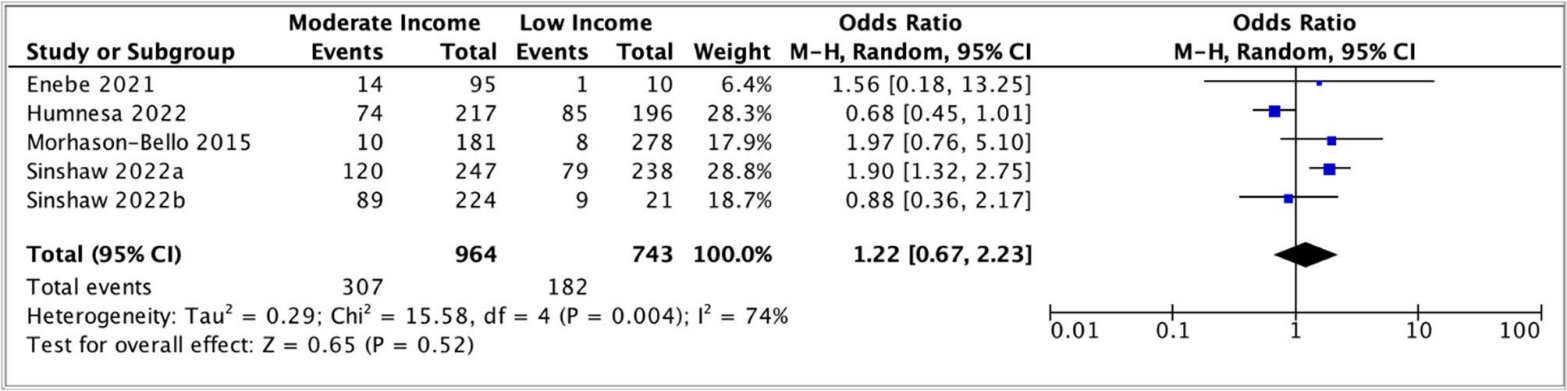
Meta-analysis of association between moderate vs. low income level and parental awareness of HPV vaccination

**Fig. 5:**
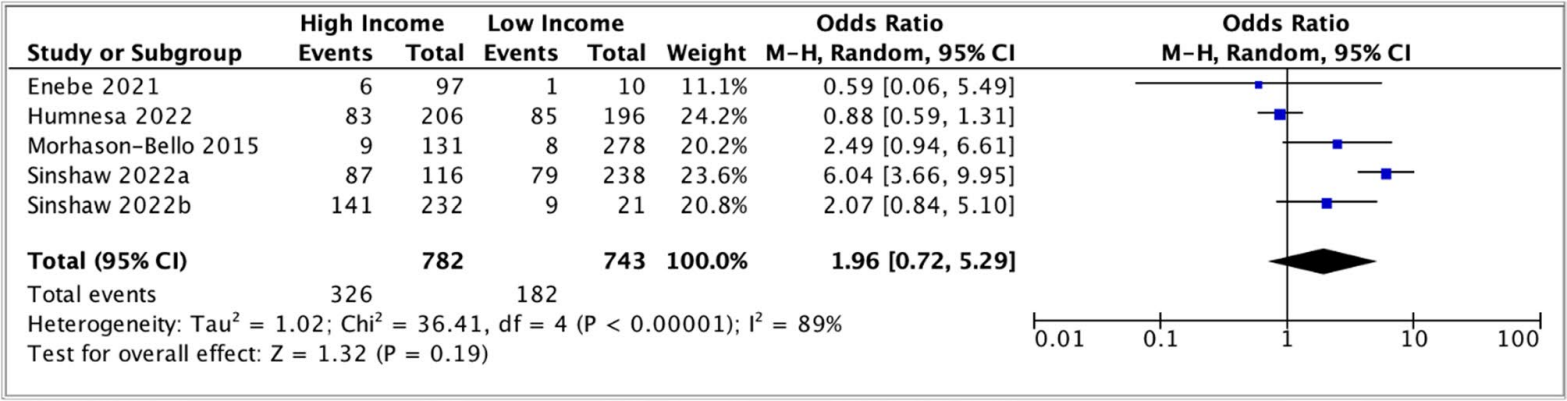
Meta-analysis of association between high vs. low-income level and parental awareness of HPV vaccination

The pooled odds ratio (1.22, 95% CI: 0.67 – 2.23) suggested a slight positive association between parental awareness and moderate income; however, the confidence interval was wide and substantial heterogeneity was observed (I 2 = 74 %) (Fig. 4). Similarly, in the meta-analysis comparing high income to low income, the pooled odds ratio was (1.96, 95% CI: 0.72 – 5.29), suggesting a positive association between higher income and parental awareness of HPV vaccination. Still, the precision of the estimates was low, and there was high heterogeneity (I 2 = 89 %) (Fig. 5).

**Fig. 6:**
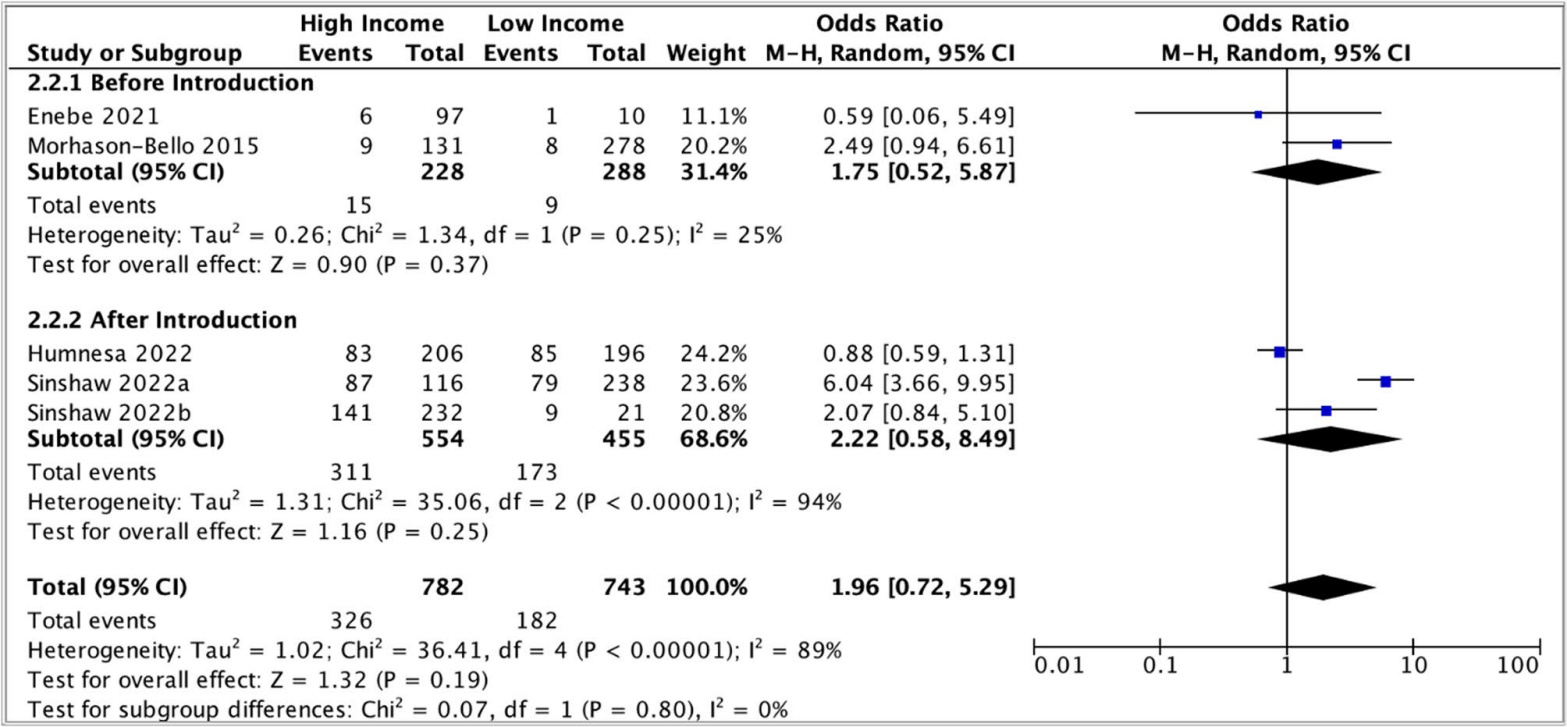
Subgroup analysis comparing association between awareness of HPV vaccination and income levels before and after the introduction of national HPV vaccination programs

Due to the substantial heterogeneity, a subgroup analysis, stratified by the timing of the study (before or after introduction of a national HPV vaccination program) was conducted (Fig. 6). The pooled odds ratios were similar in the after-introduction subgroup (2.22, 95% CI: 0.58 – 8.49) and the before-introduction subgroup (1.75, 95% CI 0.52-5.87).

Both confidence intervals were wide.

#### Association between residence and awareness

Two studies examined the association between the area of residence and awareness of HPV vaccination (*see Table S3*,* Appendix F*) [50, 62]. A study from Zimbabwe suggested a tendency towards higher awareness in rural areas (OR 1.31, 95% CI: 0.79–2.19) although the precision was low [62]. Meanwhile, a study from Ethiopia, suggested that parents in urban areas were more than four times more likely to be aware of HPV vaccination compared to parents in rural areas, with an OR of 4.23, 95% CI: 2.62–6.82 [50].

#### Association between occupation and awareness

Five studies examined the association between parents’ occupation and awareness of the HPV vaccine (*see Table S4*,* Appendix G).* Studies from Ethiopia and Nigeria found that parents with skilled occupations had higher levels of awareness of the HPV vaccine [53, 55]. Meanwhile, a study from Zimbabwe reported a tendency towards negative association between skilled occupations and awareness with an odds ratio of 0.73 (95% CI 0.31–1.72) [62]. The findings in the remaining two studies from Ethiopia were inconsistent and uncertain due to wide confidence intervals [59, 60].

### Association between socioeconomic factors and parents’ HPV vaccine acceptability

#### Association between education and acceptability of HPV vaccination

**Fig. 7:**
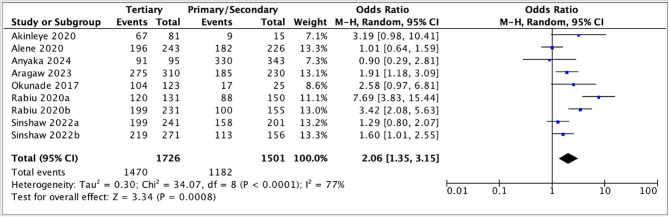
Meta-analysis of association between education and parents' willingness to vaccinate daughters against HPV

Out of the sixteen studies that examined the association between parents' education and acceptance of the HPV vaccine (Table 4), seven studies had used the predetermined education categories [30, 39–42, 58, 59] and were included in the meta-analysis (Fig. 7). In the case of two of the studies, from Nigeria and Ethiopia, estimates of association for both parents were included in the analysis [30, 59]. The odds of parents being willing to vaccinate their daughters against HPV were two times higher for those with tertiary education than those with primary/secondary education. High heterogeneity was observed between the studies (*I*^2^ = 77%).

**Table 4:** Association between education and parents’ willingness to vaccinate daughters

Author, publication year	Education	*N* (%)	OR [95% CI]	AOR [95% CI]
Akinleye et al., 2020 [39]	Primary/secondary Tertiary	9 (60.0) 67 (82.7)	Ref 3.19 (0.98–10.41)	***
Alene et al., 2020 [40]	Unable to read/write Primary/secondary Tertiary	353 (82.1) 182 (80.5) 196 (80.7)	Ref 0.90 (0.59–1.36) 0.90 (0.60–1.36)	Ref 0.66 (0.37–1.15) 0.37 (0.20–0.70)
Anyaka et al., 2024 [41]	None Primary Secondary Tertiary	63 (88.7) 144 (95.4) 186 (96.9) 91 (95.8)	Ref. 2.61 (0.91–7.52) 3.94 (1.32–11.78) 2.89 (0.83–10.01)	***
Aragaw et al., 2023 [42]	None Primary Secondary Tertiary	110 (60.8) 68 (73.1) 117 (85.4) 275 (88.7)	Ref 1.76 (1.02–3.03) 3.78 (2.16–6.61) 5.07 (3.19–8.04)	Ref 1.16 (0.59–2.28) 1.68 (0.85–3.32) 0.67 (0.27–1.72)
Destaw et al., 2021 [45]	Illiterate Primary or above	151 (68.9) 248 (87.6)	Ref 3.00 (2.02–5.03)	Ref 2.48 (1.08–6.34)
DiAngi et al., 2011 [47]	Primary or less More than primary	111 (97) 218 (85)	Ref 0.20 (0.07–0.58)	***
Kolek et al., 2022 [51]	No formal education Primary education Secondary education Tertiary education	*	0.66 (0.31–1.42) ****	0.39 (0.19–0.82) ****
Lubeya et al., 2023 [52]	None/primary Secondary/tertiary	92 (50.8) 123 (56.2)	Ref 1.24 (0.83–1.84)	***
Mihretie et al., 2022 [53]	No formal education Primary education Secondary and above	33 (18.8) 77 (33.9) 68 (28.9)	Ref 2.22 (1.39–3.55) 1.76 (1.10–2.82)	Ref 1.15 (0.34–3.44) 1.70 (1.05–2–74)
Milondzo et al., 2021 [54]	Primary education Secondary education Tertiary education Bachelors Honors Masters Doctoral	0 (0.0) 11 (16.2) 17 (13.9) 17 (18.5) 7 (13.7) 5 (23.8)	Ref 0.75 (0.32–1.78) 0.98 (0.41–2.35) 0.57 (0.20–1.63) 1.36 (0.39–4.80)	***
Ndejjo et al., 2017 [56]	None/primary Post primary	610 (93.7) 203 (91.9)	Ref 1.02 (0.81–1.27)	***
Okunade et al., 2017 [58]	Less than tertiary At least tertiary	17 (68.0) 104 (84.5)	Ref 2.58 (0.97–6.81)	***
Okunowo et al., 2021 [29]	Secondary or below Tertiary education	*	Ref 2.19 (1.13–4.25)	Ref 2.01 (0.85–4.78)
Rabiu et al., 2020 [30] (M)	None Primary Secondary Tertiary	21 (56.8) 29 (56.9) 59 (59.6) 120 (91.6)	Ref 1.00 (0.42–2–36) 1.12 (0.52–2.41) 8.31 (3.39–20.38)	***
Rabiu et al., 2020 [30] (F)	None Primary Secondary Tertiary	2 (50.0) 12 (54.5) 88 (66.2) 199 (78.8)	Ref 1.20 (0.14–10.12) 1.96 (0.27–14.34) 3.72 (0.50–27.44)	***
Sinshaw et al., 2022 [59] (M)	Cannot read and write Read and write only Primary school Secondary school Diploma Degree and above	54 (67.5) 54 (68.4) 72 (78.3) 86 (78.9) 116 (78.4) 83 (89.2)	Ref 1.04 (0.53–2.02) 1.73 (0.88–3.43) 1.80 (0.93–3.47) 1.75 (0.95–3.21) 3.99 (1.79–8.95)	Ref 0.82 (0.33–2.07) 1.37 (0.55–3.45) 1.07 (0.41–2.80) 0.91 (0.28–2.96) 1.88 (0.42–8.41)
Sinshaw et al., 2022 [59] (F)	Cannot read and write Read and write only Primary school Secondary school Diploma Degree and above	8 (66.7) 26 (68.4) 49 (70.0) 64 (74.4) 94 (75.8) 125 (85.0)	Ref 1.08 (0.27–4.31) 1.17 (0.32–4.30) 1.46 (0.40–5.31) 1.57 (0.44–5.57) 2.84 (0.79–10.25)	Ref 0.87 (0.19–4.12) 0.87 (0.19–4.05) 0.82 (0.18–3.63) 0.68 (0.14–3.20) 0.80 (0.15–4.22)
Vermandere et al., 2014 [61]	Years of education	*	**	1.05 (1.01–1.08)

*(M) indicates mothers*,* (F) indicates fathers*

* Information on N (%) was missing

** information on OR was missing

*** information on AOR was missing

**** Only combined odds ratios were provided,* possibly to address challenges related to small sample sizes associated with the specific education levels*

**Fig. 8:**
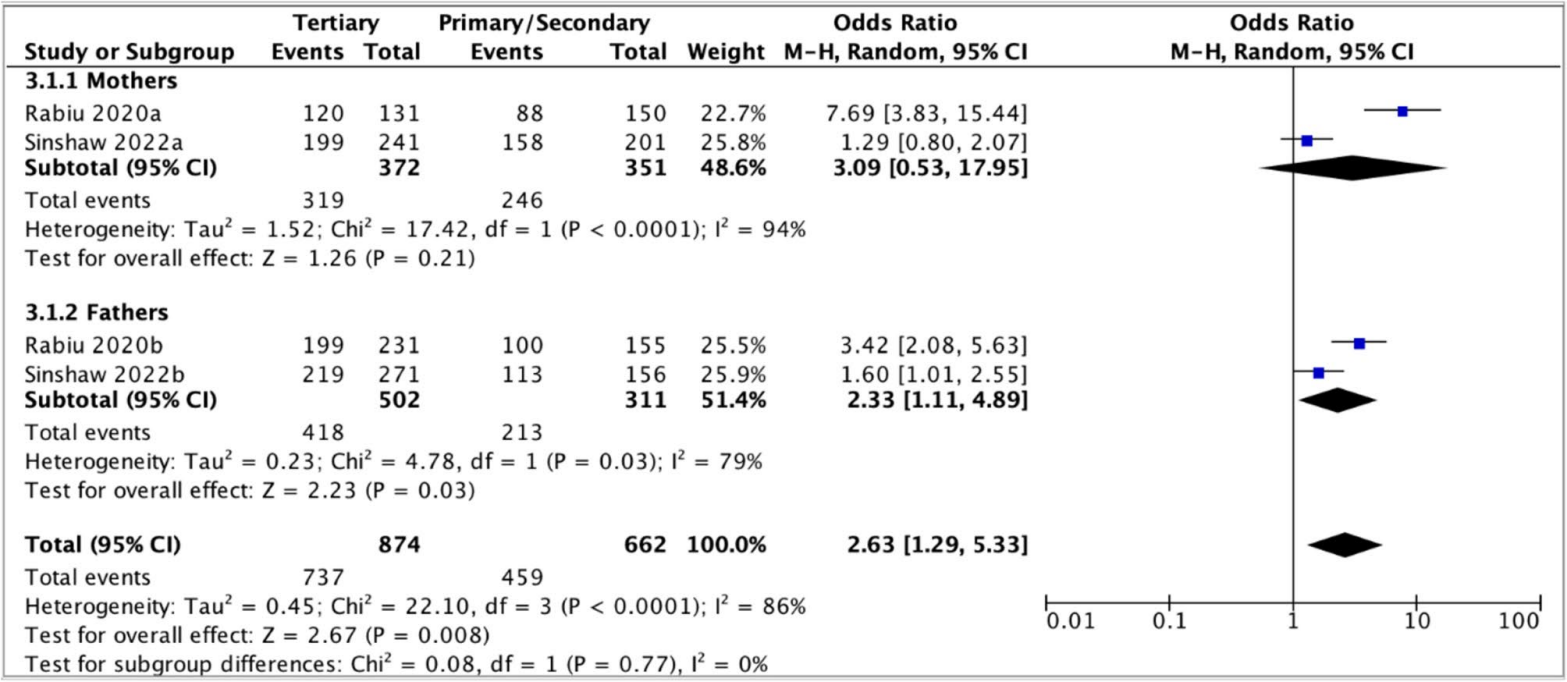
Subgroup analysis comparing willingness to vaccinate and its association with parental education among mothers and fathers

To investigate the high heterogeneity, subgroup analyses stratifying on parents’ gender and the timing of the study were conducted (Fig. 8). The pooled odds ratio for those with tertiary versus primary and secondary education were similar among mothers and fathers (OR 3.09, 95% CI: 0.53 – 17.95 versus OR 2.33, 95% CI: 1.11 – 4.89). The wide confidence intervals overlapped, and the estimate for mothers was highly uncertain. Additionally, there was high heterogeneity in both subgroups. Although the estimates are of similar magnitude, it's not certain that one is larger than the other.

**Fig. 9:**
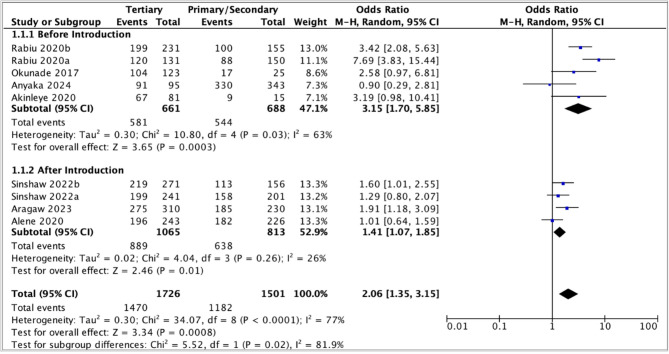
Subgroup analysis comparing willingness to vaccinate and its association with parental education before and after the introduction of national HPV vaccination programs

The subgroup analysis, based on the timing of the study, indicated that the pooled odds ratio for parents with tertiary education versus those with primary/secondary education was higher in studies conducted before the introduction of national HPV vaccination programs (3.15, 95% CI: 1.70 – 5.85) than in those conducted after the introduction (1.41, 95% CI 1.07 – 1.85) (Fig. 9).

The remaining studies revealed varying associations between education and parental acceptance of the HPV vaccine. Parents with formal education showed a higher willingness to vaccinate their daughters, according to four studies, two conducted in Ethiopia, one in Nigeria, and one in Kenya [29, 45, 53, 61]. In contrast, studies from Botswana and Kenya reported that parents with higher education levels were less willing to vaccinate their daughters against HPV [47, 51]. Meanwhile, studies from South Africa and Uganda found no clear association [54, 56].

**Fig. 10:**
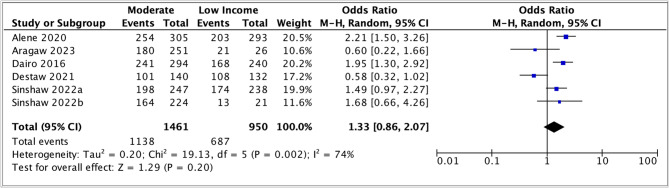
Meta-analysis of association between moderate income vs low income and parental acceptance of HPV vaccination

Nine studies investigated the association between income and parents' willingness to vaccinate their daughters against HPV (see Table S5, Appendix H) [40, 42, 44–47, 52, 56, 59]. Five of these were included in the meta-analysis. The pooled odds ratio (1.33, 95% CI: 0.86 – 2.07) suggested that parents with moderate income showed slightly higher levels of acceptance of the HPV vaccine (Fig. 10), but there was some uncertainty regarding the estimate. The heterogeneity was high (*I*^2^ = 74%).

**Fig. 11:**
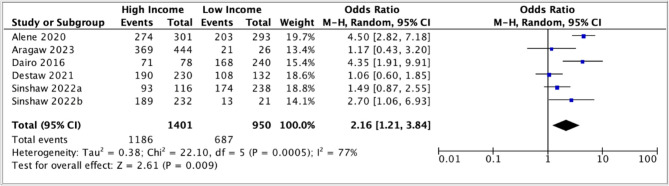
Meta-analysis of association between high income vs low income and parental acceptance of HPV vaccination

In the meta-analysis comparing high income to low income, the pooled odds ratio was (2.16, 95% CI: 1.21 – 3.84) (Fig. 11). High heterogeneity (*I*^2^ = 77%) was observed in this analysis too.

#### Association between residence and acceptability

**Fig. 12:**
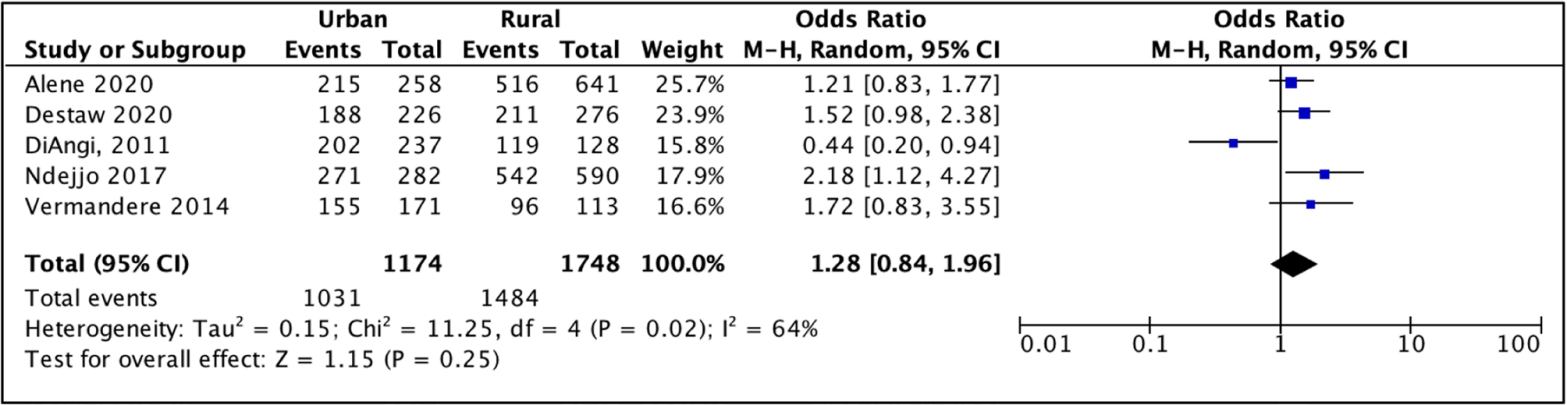
Meta-analysis of association between area of residence and parents' willingness to vaccinate daughters against HPV

Five studies examined the effect of area of residence on HPV vaccine acceptability (*see Table S6*,* Appendix I*). Four of the included studies suggested higher acceptability among parents living in urban areas [40, 45, 56, 61]. Meanwhile, a study conducted in Botswana indicated that there was a negative association between urban residence and acceptability [47] but it should be noted that this study was assessed to have a high risk of bias. Overall, the pooled odds ratio (1.28, 95% CI: 0.84–1.96) suggested a slight positive association between parents living in urban areas and their acceptance of the HPV vaccine (Fig. 12). However, there was substantial uncertainty and heterogeneity observed (*I*^*2*^ = 64%).

#### Association between occupation and acceptability

Ten studies investigated the relationship between parents’ occupational level and their acceptability of the HPV vaccine (*see Table S7*,* Appendix J).* In studies from Nigeria, South Africa and Ethiopia, it was found that parents with skilled occupations showed more willingness to vaccinate their daughters against HPV [29, 30, 42, 44, 54]. Another study conducted in Ethiopia found that the willingness to vaccinate among parents working as government employees was similar to that of other occupations, including self-employed, farmers, merchants, and daily laborers [53]. Studies from Kenya and Uganda found no important association (OR 0.92, 95% CI: 0.48–1.76 [51] and OR 0.94. 95% CI: 0.91–0.98) [56], respectively).

### Certainty of evidence

The GRADE assessment of the four meta-analyses is summarized in Table 5, where risk of bias, inconsistency, imprecision, indirectness, and publication bias were examined. The explanations for the assessment are provided in the supplementary file (*see Appendix L).*

**Table 5:** GRADE assessment of meta-analyses

No. of participants (studies)	Risk of bias	Inconsistency	Imprecision	Indirectness	Publication bias	Quality of evidence (GRADE)
Association between education and acceptability of HPV vaccination						
3497 (7 studies)	Some concerns	Serious inconsistency	Not serious	Serious indirectness	Not serious	Low ⊕⊕ΟΟ
Association between education and parental awareness of HPV vaccination						
2019 (4 studies)	Some concerns	Serious inconsistency	Serious imprecision	Serious indirectness	Serious	Very low ⊕ΟΟΟ
Association between income and acceptability of HPV vaccination (high income vs. low income)						
3335 (5 studies)	Some concerns	Serious inconsistency	Not serious	Serious indirectness	Not serious	Low ⊕⊕ΟΟ
Association between income and acceptability of HPV vaccination (moderate income vs. low income)						
3335 (5 studies)	Some concerns	Serious inconsistency	Not serious	Serious indirectness	Not serious	Low ⊕⊕ΟΟ
Association between income and awareness of HPV (high income vs. low income)						
2599 (4 studies)	Some concerns	Serious inconsistency	Serious imprecision	Serious indirectness	Not serious	Very low ⊕ΟΟΟ
Association between income and awareness of HPV (moderate income vs. low income)						
2599 (4 studies)	Some concerns	Serious inconsistency	Not serious	Serious indirectness	Not serious	Low ⊕⊕ΟΟ
Association between residence and acceptability						
2064 (5 studies)	Some concerns	Serious inconsistency	Not serious	Serious indirectness	Not serious	Low ⊕⊕ΟΟ

The evidence for the associations between education and acceptability, high-income and acceptability, moderate-income and acceptability, moderate income and awareness, and residence and acceptability was deemed to have a low certainty. The evidence for the association between education and awareness, and high-income levels and awareness was deemed to have very low certainty.

## Discussion

A total of 26 studies were examined, including eight studies that focused solely on awareness of the HPV vaccine, 14 studies that focused solely on acceptability, and four studies that assessed both outcomes. Higher education was found to be associated with higher awareness in most studies, although some inconsistencies were observed across the studies. The meta-analysis investigating the relationship between income and awareness of HPV vaccination indicated that parents with higher and moderate income possibly had higher odds of being aware of HPV vaccination than those with lower income, but the precision was low. Furthermore, parents with skilled occupations have shown higher levels of awareness of the HPV vaccine in several studies, although there were conflicting findings regarding this association. There were too few studies to conclude regarding the association between residence and awareness. A meta-analysis investigating the relationship between education and acceptance found that parents with higher educational levels showed higher willingness to vaccinate their daughters against HPV. Similarly, a positive association was found between high monthly income and acceptability of the HPV vaccine. While some studies reported that parents with skilled occupations tended to show greater willingness to accept the HPV vaccine for their children, the evidence was mixed and not consistent across all studies. For residence, a meta-analysis suggested a possible positive association between urban residence and acceptability; however, there was substantial uncertainty around the estimate. Overall, while socioeconomic factors appeared to play a role in HPV vaccine acceptability and awareness, the associations were not consistent across all studies.

Our finding that the majority of the studies from SSA indicate a correlation between higher levels of education and both parental awareness and acceptance of the HPV vaccine is consistent with studies conducted in China [63], Vietnam [64] and Latin America [65] and a systematic review involving European adolescents and parents [66]. According to the latter, receiving information on vaccines from healthcare providers was linked to increased awareness and acceptance among both parents and adolescents, again linked to higher education [66]. This may indicate that individuals with higher education may have greater trust in healthcare professionals, greater access to information related to health [33], and higher health literacy, which could result in increased exposure to information about HPV. This in turn may lead to a greater understanding of the risks associated with HPV infections, as well as the importance of prioritizing preventive healthcare.

In our review, the association between education and parental acceptance was found to be higher in studies conducted before the introduction of national HPV vaccination programs. Parents with lower education levels were likely much less exposed to information about HPV vaccines during this period. Meanwhile, after the introduction of national HPV programs, the increased sensitization and exposure of most parents to information about the HPV vaccine, may have resulted in the association between education and parental acceptance becoming less pronounced.

The tendency towards a positive association between higher monthly income and parental awareness and acceptability of the HPV vaccine could possibly be attributed to increased access to healthcare facilities, greater financial means, and better access to media and educational resources [67]. One study conducted in Nigeria before the introduction of national HPV vaccination found that the high cost of the HPV vaccine significantly influenced parents’ willingness to vaccinate their daughters [39]. This highlights the critical need for affordable or free HPV vaccines to ensure that all households have equal access to them, regardless of their financial situation. It is worth noting that some of the studies included in the meta-analysis had relatively small sample sizes, which contributed to imprecise estimates.

The overall inconsistent findings regarding occupation and parents’ awareness and acceptance of the HPV vaccine may be attributed to the varying definitions of occupation used across studies. While some studies specified job titles, others distinguished between skilled and unskilled workers, and some differentiated based on employment status. This made it difficult to make meaningful comparisons across studies and find clear associations. Additionally, some studies had small samples of certain occupational categories, which could have further contributed to the mixed results.

For the relationship between residence and parents’ vaccine willingness to vaccinate, the majority of studies found that parents in urban areas were more likely to be willing to vaccinate their daughters. It is likely that urban residents have greater access to healthcare or are more exposed to health campaigns compared to parents in rural areas, and thus were more willing to vaccinate their daughters. Research has suggested that individuals residing in urban areas may be less likely to be misinformed regarding vaccines [68], which could lead to less hesitancy and more willingness.

Based on the GRADE assessment, there was very low or low certainty of evidence across the different meta-analyses, partly due to the limited number of studies identified. Overall, given the current limitations in the evidence, future research should explore socioeconomic disparities in HPV vaccination status across Sub-Saharan Africa, as most countries in the region have now implemented national HPV vaccination programs.

This is the first systematic to examine the relationships between socioeconomic factors and parents’ awareness and acceptance of HPV vaccination in SSA. Bias was minimized by having three reviewers conduct the screening of studies and the extraction of data independently. A qualified librarian was also consulted to develop and execute a comprehensive search strategy across multiple databases. However, the review also has some limitations. The findings may have limited generalizability due to the inclusion of only seven of the forty-six countries in SSA. The findings from studies conducted before the introduction of national HPV vaccination programs cannot necessarily be extrapolated to countries that have introduced the program, nor vice versa. The included studies may also have been subjected to selection bias. One of the included studies was conducted in a hospital setting and predominantly included individuals with higher educational levels compared to the general population [58], while another study focused on urban respondents only [51]. As a result, the findings may be less generalizable to the general population, especially those living in rural areas. Another limitation is the potential for information bias. The primary tool for data collection in the included studies was questionnaires. Studies that used non-standardized or non-validated questionnaires may have been particularly prone to bias. Additionally, as the collected data were mainly self-reported, the risk of information bias may have been further enhanced. For instance, there may be recall bias when parents reported on the vaccination status of their daughters. Parents may also be prone to social desirability bias, such as over-reporting awareness or willingness to vaccinate and under-reporting negative attitudes towards the vaccine. Lastly, as only English studies were included in this review, there may be an incomplete representation of available evidence provided in studies published in other languages.

While conducting the meta-analyses, it became apparent that the studies had high heterogeneity. Despite random effects models and subgroup analyses, substantial heterogeneity remained in some of the analyses. This suggests that there were other unmeasured factors and sources of variability that were unaccounted for. Given the low number of included studies in each meta-analysis and that a considerable number of the included studies had relatively small sample sizes, the obtained pooled estimates were less precise than desirable. As a result, the generalizability of the findings across sub-Saharan African countries may be limited, especially given the variation in social structures, cultural norms, and healthcare systems across the region.

As our findings suggest a positive association between higher education and income with parents’ awareness and acceptance of the HPV vaccine, it emphasizes the importance of considering socioeconomic differences when implementing national HPV vaccination programs in sub-Saharan Africa. Neglecting these differences may exacerbate existing health disparities. By accounting for socioeconomic differences and addressing them in the development of vaccination programs, we could potentially achieve equitable vaccine distribution and counter the suspected inequities in cervical cancer.

Including HPV vaccination as part of children’s routine immunization in SSA may be an effective approach to address vaccine hesitancy and increase coverage. The absence of the HPV vaccine from routine immunization programs may contribute to parental suspicions. A study conducted in the Netherlands found that parents perceived the HPV vaccine as different in comparison to other childhood vaccines, resulting in negative attitudes and vaccine hesitancy [69]. Research conducted in Japan suggested that incorporating vaccines into routine immunization programs could reduce parental vaccine hesitancy by building trust [70].

The introduction of school-based vaccination delivery may be an effective strategy to increase vaccine coverage. The success of this approach has been demonstrated in numerous countries, including Rwanda [71], Zimbabwe [72], Malaysia [73] and Australia [74]. By implementing school-based vaccination programs, a larger number of children can be reached, while possibly overcoming barriers such as cost and transportation. This approach may also be effective in reaching children who live in areas with less access to healthcare facilities, making it a convenient option for parents.

Efforts to reduce cost and accessibility barriers are necessary for ensuring equitable access to healthcare services. The cost of vaccinating can be a challenge for parents, as demonstrated by a study in Nigeria where the majority of parents were unwilling to pay for the HPV vaccine due to financial concerns [75]. Furthermore, according to a systematic review, high transportation cost due to great travel distance was one of the factors contributing to incomplete vaccination in SSA [20]. To address this issue, the provision of mobile clinics or outreach services may be a viable strategy to improve vaccine coverage in underserved areas. In Cameroon, this strategy was found to increase vaccine uptake among girls living in harder-to-reach areas [76]. According to Watson-Jones et al., mobile-outreach activities should be implemented alongside school-based delivery to ensure that out-of-school girls living in hard-to-reach communities are reached [77].

## Conclusion

This systematic review suggests that parents’ awareness and acceptance of HPV vaccination in SSA may be positively associated with socioeconomic status, specifically higher education, income levels and urban residence. The association between education and parental acceptance of the HPV vaccine tended to be stronger in studies conducted before the introduction of national HPV immunization programs. The associations between occupation and parents’ awareness and acceptance, as well as the association between residence and awareness, were inconsistent. Despite conducting subgroup analyses to investigate potential sources of heterogeneity, the remaining unexplained heterogeneity suggests that there were other underlying factors contributing to the varying associations. While this systematic review provides valuable insight into the association between socioeconomic factors and HPV vaccine awareness and acceptability in SSA, it is important to acknowledge that the data were collected from a limited number of countries within the region and may therefore have limitations regarding generalizability. Overall, this systematic review indicates the need to consider socioeconomic differences in awareness and acceptability when scaling up HPV vaccination, to avoid reinforcing existing socioeconomic disparities in the prevalence of HPV and cervical cancer.

## Supplementary Information

Supplementary Material 1.

## Data Availability

All data is provided within the manuscript or the supplementary file.

## Abbreviations

HPV: Human papillomavirus
STI: Sexually transmitted infections
WHO: World Health Organization
LMICs: Low- and middle-income countries
SSA: sub-Saharan Africa
FDA: Food and Drug Administration
GAVI: Gavi, the Vaccine Alliance
PRISMA: Preferred Reporting Items for Systematic Reviews and Meta-Analyses
MeSH: Medical Subject Headings
AOR: Adjusted odds ratio
CI: Confidence interval
OR: Odds ratio
JBI: Joanna Briggs Institute
GRADE: Grading of Recommendations, Assessment, Development and Evaluation
