# Supplementary material for: Associations of socioeconomic factors with parents’ awareness and acceptability of HPV vaccination in sub-Saharan Africa - a systematic review and meta-analysis
Source: BMC Public Health. 2025 Oct 17;25:3525. doi: 10.1186/s12889-025-24550-x (PMC12535123; doi:10.1186/s12889-025-24550-x)
Supplement: Supplementary file 1 — Supplementary Material 1. [file 12889_2025_24550_MOESM1_ESM.docx]

SUPPLEMENTARY MATERIAL

**Appendix A** – Search strategies in different databases

## Appendix B – JBI checklist for critical appraisal of cross-sectional studies

## Appendix C – JBI checklist for critical appraisal of cohort studies

**Appendix D** – Risk of bias assessment of Cross-Sectional Studies (Table S1)

**Appendix E** – Risk of bias assessment of Cohort Studies (Table S2)

**Appendix F** – Association between residence and parents’ awareness (Table S3)

**Appendix G** – Association between occupation and parents’ awareness (Table S4)

**Appendix H** - Association between income and parents’ willingness to vaccinate daughters (Table S5)

**Appendix I** – Association between residence and parents’ willingness to vaccinate daughters (Table S6)

**Appendix J** – Association between occupation and parents’ willingness to vaccinate daughters (Table S7)

**Appendix K** – GRADE Assessment - Funnel plots

**Appendix L** – GRADE Assessment - Explanations

**Appendix M** – PRISMA 2020 Checklist

**Appendix A – Search strategies in different databases**

Search filters applied in all four databases

Language: English

Year: 2000-2022

PUBMED

Number of hits: 245

("Papillomavirus Vaccines"[mesh] OR "Papillomavirus Vaccines"[nm]) OR "Papillomavirus Infections"[mesh] OR "Papillomaviridae"[mesh] OR alphapapilloma*[tiab] OR betapapilloma*[tiab] OR gammapapilloma*[tiab] OR mupapilloma*[tiab] OR papilloma*[tiab] OR HPV[tiab] or “human papillomavirus*”[tiab] OR “wart virus”[tiab] OR “genital wart*”[tiab]

AND

(vaccine*[tiab] OR vaccination*[tiab] OR immuniz*[tiab] OR immunis*[tiab] OR gardasil[tiab] OR cervarix[tiab] OR "Immunization"[mesh] OR "Immunization Programs"[mesh])

AND

(“socioeconomic factors”[all fields] OR “education”[all fields] OR "educational status"[all fields] OR “income”[all fields] OR "economic status"[all fields] OR "economic stability" [all fields] OR "Salaries and Fringe Benefits"[mesh] OR “occupation*”[all fields] OR “employment”[all fields] OR "social class"[all fields] OR “unemployment”[all fields] OR “social status”[all fields] OR “workplace”[all fields] OR “household expenditure”[all fields])

AND

("parent*"[all fields] OR "guardian*"[all fields] OR "legal guardians"[all fields] OR “caregiver*”[all fields] OR “mother*”[all fields] OR woman*[all fields] OR women*[all fields] OR “father*”[all fields] OR daughter*[all fields] OR “eligible daughter*”[all fields] OR girl*[all fields] OR child*[all fields])

AND

("Africa South of the Sahara"[Mesh] OR Angola*[tiab] OR Benin*[tiab] OR Botswana*[tiab] OR “Burkina Faso”[tiab] OR Burundi*[tiab] OR "Cabo Verde*"[tiab] OR Cameroon*[tiab] OR “Central African Republic”[tiab] OR Chad*[tiab] OR Comoros[tiab] OR “Democratic Republic of Congo”[tiab] OR “Cote d'Ivoire”[tiab] OR Equatorial Guinea[tiab] OR Eritrea*[tiab] OR Eswatini[tiab] OR Ethiopia*[tiab] OR Gabon*[tiab] OR Gambia*[tiab] OR Ghana*[tiab] OR Guinea*[tiab] OR “Guinea Bissau”[tiab] OR “Ivory Coast”[tiab] OR Kenya*[tiab] OR Lesotho[tiab] OR Liberia*[tiab] OR Madagascar[tiab] OR Malawi*[tiab] OR Mali*[tiab] OR Mauritania*[tiab] OR Mauritius[tiab] OR Mozambique[tiab] OR Namibia*[tiab] OR Niger*[tiab] OR Nigeria*[tiab] OR Rwanda*[tiab] OR “Sao Tome and Principe”[tiab] OR Senegal*[tiab] OR Seychelles[tiab] OR "Sierra Leone*"[tiab] OR Somalia*[tiab] OR "South Africa*"[tiab] OR "South Sudan*"[tiab] OR Sudan*[tiab] OR Swaziland[tiab] OR Tanzania*[tiab] OR Togo*[tiab] OR Uganda*[tiab] OR Zambia*[tiab] OR Zimbabwe*[tiab])

EMBASE

Number of hits: 40

exp papillomavirus infection/ or exp Papilloma virus/ or exp Papillomaviridae/

OR (alphapapilloma* or betapapilloma* or gammapapilloma* or mupapilloma* or papilloma* or HPV or wart virus or genital wart*).mp.

AND

exp Human papilloma virus vaccine/ or exp immunization/ or exp preventive health service/

OR (vaccine* or vaccination* or immuniz* or immunis* or gardasil or cervarix).mp.

AND

(Salaries and Fringe Benefits or household expenditure).mp.

exp socioeconomics/ or exp health education/ or exp education/ or exp educational status/ or exp income inequality/ or exp personal income/ or exp household income/ or exp income security/ or exp income distribution/ or exp family income/ or exp income/ or exp income group/ or exp social class/ or exp economic status/ or exp social status/ or exp occupation/ or exp employment status/ or exp employment/ or exp parental employment status/ or exp unemployment/ or exp workplace/

AND

exp parent/ or exp legal guardians/ or exp caregiver/ or exp mother*/ or exp father*/ or exp women*/ or exp women*/ or exp daughter*/ or exp child*/ or exp girl*/

AND

exp "Africa south of the Sahara"/ or (Angola* OR Benin* OR Botswana* OR Burkina Faso OR Burundi* OR "Cabo Verde*" OR Cameroon* OR Central African Republic OR Chad* OR Comoros OR Democratic Republic of Congo OR Cote d'Ivoire OR Equatorial Guinea OR Eritrea* OR Eswatini OR Ethiopia* OR Gabon* OR Gambia* OR Ghana* OR Guinea* OR Guinea Bissau OR Kenya* OR Lesotho OR Liberia* OR Madagascar OR Malawi* OR Mali* OR Mauritania* OR Mauritius OR Mozambique OR Namibia* OR Niger* OR Nigeria* OR Rwanda* OR Sao Tome and Principe OR Senegal* OR Seychelles OR Sierra Leone* OR Somalia* OR South Africa* OR South Sudan* OR Sudan* OR Swaziland OR Tanzania* OR Togo* OR Uganda* OR Zambia* OR Zimbabwe*).mp.

WEB OF SCIENCE

Number of hits: 234

(TS=(Papillomavirus Infections OR Papillomaviridae OR alphapapilloma* OR betapapilloma* OR gammapapilloma* OR mupapilloma* OR papilloma* OR HPV OR “wart virus” OR “genital wart*”)

AND

TS=(Papillomavirus Vaccines OR Immunity OR Immunization Programs OR vaccine* OR vaccination* OR immuniz* OR immunis* OR gardasil OR cervarix)

AND

ALL=(“socioeconomic factors” OR “education” OR "educational status" OR “income” OR "economic status" OR "economic stability" OR "Salaries and Fringe Benefits" OR “occupations” OR “employment” OR "social class" OR “unemployment” OR “social status” OR “workplace” OR “household expenditure”)

AND

ALL=("parent*" OR "guardian*" OR "legal guardians" OR “caregiver*” OR “mother*” OR “father*” OR “daughter*” OR “child*” OR “girl*”)

AND

TS=("Africa South of the Sahara" OR Africa* OR Angola* OR Benin* OR Botswana* OR Burkina Faso OR Burundi* OR "Cabo Verde*" OR Cameroon* OR Central African Republic OR Chad* OR Comoros OR Democratic Republic of Congo OR Cote d'Ivoire OR Equatorial Guinea OR Eritrea* OR Eswatini OR Ethiopia* OR Gabon* OR Gambia* OR Ghana* OR Guinea* OR Guinea Bissau OR Kenya* OR Lesotho OR Liberia* OR Madagascar OR Malawi* OR Mali* OR Mauritania* OR Mauritius OR Mozambique OR Namibia* OR Niger* OR Nigeria* OR Rwanda* OR “Sao Tome and Principe” OR Senegal* OR Seychelles OR "Sierra Leone*" OR Somalia* OR "South Africa*" OR "South Sudan*" OR Sudan* OR Swaziland OR Tanzania* OR Togo* OR Uganda* OR Zambia* OR Zimbabwe*))

COCHRANE

Number of hits: 7

Papillomavirus Infections[mesh]

OR Papillomaviridae[mesh])

OR (alphapapilloma* OR betapapilloma* OR gammapapilloma* OR mupapilloma* OR papilloma* OR HPV OR “wart virus” OR “genital wart*”):ti,ab,kw

AND

Papillomavirus Vaccines[mesh]

OR Immunity[mesh]

OR Immunization Programs[mesh])

OR (vaccine* OR vaccination* OR immuniz* OR immunis* OR gardasil OR cervarix):ti,ab,kw

AND

Attitude to Health[mesh]

OR Patient Acceptance of Health Care[mesh]

OR (“accept*” OR acceptance OR acceptability OR knowledge OR “aware*” OR “awareness” OR belief* OR attitude* OR perception* OR understanding* OR adherence* OR compliance OR uptake OR “willing*” OR “hesit*” OR practice*)

AND

Socioeconomic factors[mesh]

OR education[mesh]

OR educational status[mesh]

OR “income”[mesh]

OR "economic status"[mesh]

OR "Salaries and Fringe Benefits"[mesh]

OR “occupations”[mesh]

OR “employment”[mesh]

OR “unemployment”[mesh]

OR "social class"[mesh]

OR “social status”[mesh]

OR “workplace”[mesh]

OR “household expenditure”

AND

(“socioeconomic factors" OR “education” OR "educational status" OR “income” OR "economic status" OR "economic stability" OR “occupation*” OR “employment” OR "social class" OR “unemployment” OR “social status” OR “workplace” OR “household expenditure”)

AND

"parenting"[mesh]

("parent*" OR "guardian*" OR "legal guardians" OR “caregiver*” OR “mother*” OR “father*” OR “daughter*” OR “child*” OR “girl*”):ti,ab,kw

AND

("Africa South of the Sahara"[Mesh]) OR (Africa* OR Angola* OR Benin* OR Botswana* OR Burkina Faso OR Burundi* OR "Cabo Verde*" OR Cameroon* OR Central African Republic OR Chad* OR Comoros OR Democratic Republic of Congo OR Cote d'Ivoire OR Equatorial Guinea OR Eritrea* OR Eswatini OR Ethiopia* OR Gabon* OR Gambia* OR Ghana* OR Guinea* OR Guinea Bissau OR Kenya* OR Lesotho OR Liberia* OR Madagascar OR Malawi* OR Mali* OR Mauritania* OR Mauritius OR Mozambique OR Namibia* OR Niger* OR Nigeria* OR Rwanda* OR “Sao Tome and Principe” OR Senegal* OR Seychelles OR "Sierra Leone*" OR Somalia* OR "South Africa*" OR "South Sudan*" OR Sudan* OR Swaziland OR Tanzania* OR Togo* OR Uganda* OR Zambia* OR Zimbabwe*):ti,ab,kw

## Appendix B – JBI checklist for critical appraisal of cross-sectional studies

Answers: Yes, No, Unclear or Not/Applicable

**1. Were the criteria for inclusion in the sample clearly defined?**

The authors should provide clear inclusion and exclusion criteria that they developed prior to recruitment of the study participants. The inclusion/exclusion criteria should be specified (e.g., risk, stage of disease progression) with sufficient detail and all the necessary information critical to the study.

**2. Were the study subjects and the setting described in detail?**

The study sample should be described in sufficient detail so that other researchers can determine if it is comparable to the population of interest to them. The authors should provide a clear description of the population from which the study participants were selected or recruited, including demographics, location, and time period.

**3. Was the exposure measured in a valid and reliable way?**

The study should clearly describe the method of measurement of exposure. Assessing validity requires that a 'gold standard' is available to which the measure can be compared. The validity of exposure measurement usually relates to whether a current measure is appropriate or whether a measure of past exposure is needed. Reliability refers to the processes included in an epidemiological study to check repeatability of measurements of the exposures. These usually include intra-observer reliability and inter-observer reliability.

**4. Were objective, standard criteria used for measurement of the condition?**

It is useful to determine if patients were included in the study based on either a specified diagnosis or definition. This is more likely to decrease the risk of bias. Characteristics are another useful approach to matching groups, and studies that did not use specified diagnostic methods or definitions should provide evidence on matching by key characteristics

**5. Were confounding factors identified?**

Confounding has occurred where the estimated intervention exposure effect is biased by the presence of some difference between the comparison groups (apart from the exposure investigated/of interest). Typical confounders include baseline characteristics, prognostic factors, or concomitant exposures (e.g. smoking). A confounder is a difference between the comparison groups and it influences the direction of the study results. A high quality study at the level of cohort design will identify the potential confounders and measure them (where possible). This is difficult for studies where behavioral, attitudinal or lifestyle factors may impact on the results.

**6. Were strategies to deal with confounding factors stated?**

Strategies to deal with effects of confounding factors may be dealt within the study design or in data analysis. By matching or stratifying sampling of participants, effects of confounding factors can be adjusted for. When dealing with adjustment in data analysis, assess the statistics used in the study. Most will be some form of multivariate regression analysis to account for the confounding factors measured.

**
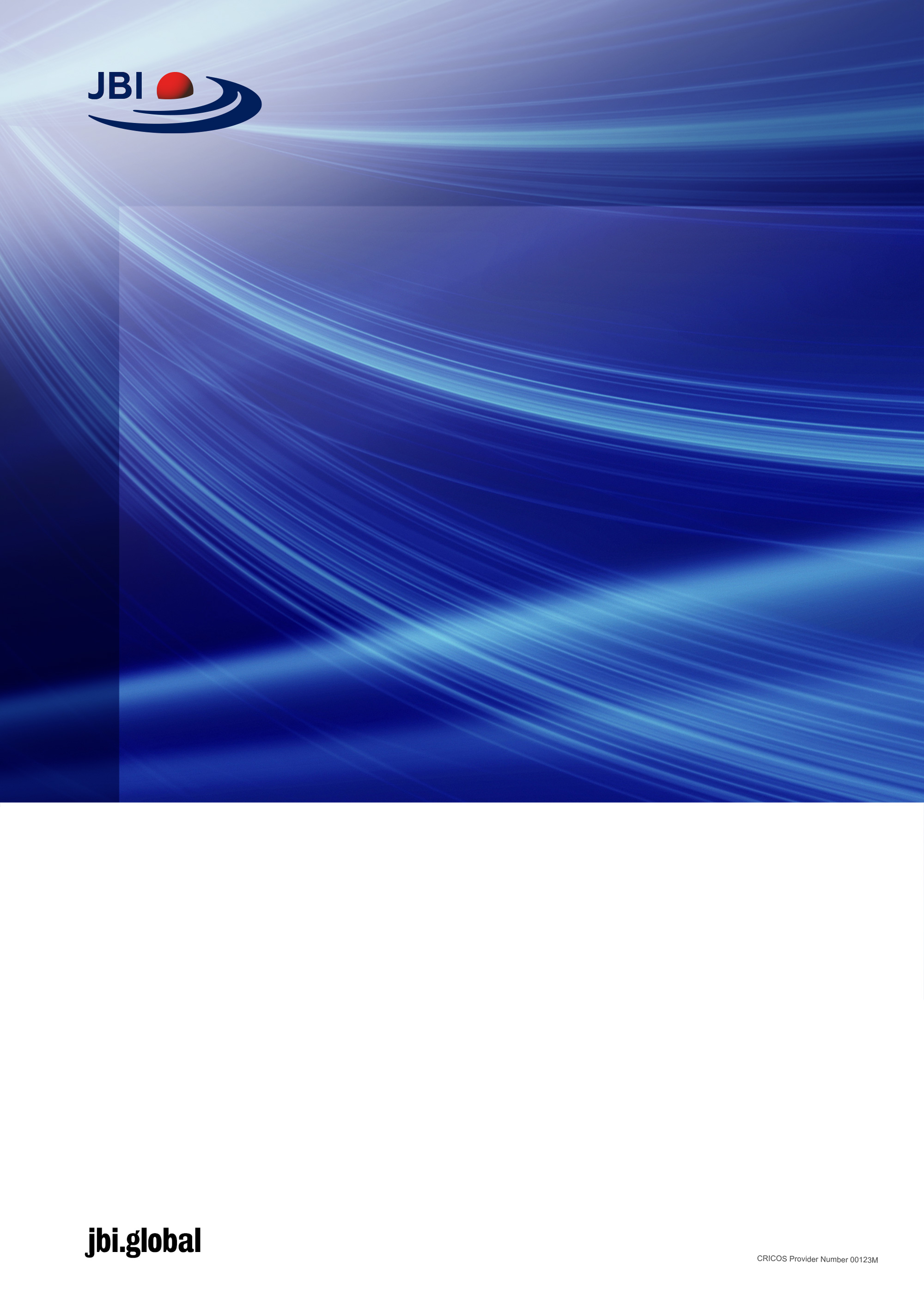
7. Were the outcomes measured in a valid and reliable way?**

Read the methods section of the paper. If for e.g. lung cancer is assessed based on existing definitions or diagnostic criteria, then the answer to this question is likely to be yes. If lung cancer is assessed using observer reported, or self-reported scales, the risk of over- or under-reporting is increased, and objectivity is compromised. Importantly, determine if the measurement tools used were validated instruments as this has a significant impact on outcome assessment validity. Having established the objectivity of the outcome measurement (e.g. lung cancer) instrument, it’s important to establish how the measurement was conducted. Were those involved in collecting data trained or educated in the use of the instrument/s? (e.g. radiographers). If there was more than one data collector, were they similar in terms of level of education, clinical or research experience, or level of responsibility in the piece of research being appraised?

**8. Was appropriate statistical analysis used?**

As with any consideration of statistical analysis, consideration should be given to whether there was a more appropriate alternate statistical method that could have been used. The methods section should be detailed enough for reviewers to identify which analytical techniques were used (in particular, regression or stratification) and how specific confounders were measured. For studies utilizing regression analysis, it is useful to identify if the study identified which variables were included and how they related to the outcome. If stratification was the analytical approach used, were the strata of analysis defined by the specified variables? Additionally, it is also important to assess the appropriateness of the analytical strategy in terms of the assumptions associated with the approach as differing methods of analysis are based on differing assumptions about the data and how it will respond.

Moola S, Munn Z, Tufanaru C, Aromataris E, Sears K, Sfetcu R, Currie M, Qureshi R, Mattis P, Lisy K, Mu P-F. Chapter 7: Systematic reviews of etiology and risk . In: Aromataris E, Munn Z (Editors). JBI Manual for Evidence Synthesis. JBI, 2020. Available from <https://synthesismanual.jbi.global>

## Appendix C – JBI checklist for critical appraisal of cohort studies

**1. Were the two groups similar and recruited from the same population?**

Check the paper carefully for descriptions of participants to determine if patients within and across groups have similar characteristics in relation to exposure (e.g. risk factor under investigation). The two groups selected for comparison should be as similar as possible in all characteristics except for their exposure status, relevant to the study in question. The authors should provide clear inclusion and exclusion criteria that they developed prior to recruitment of the study participants.

**2. Were the exposures measured similarly to assign people to both exposed and unexposed groups?**

A high quality study at the level of cohort design should mention or describe how the exposures were measured. The exposure measures should be clearly defined and described in detail. This will enable reviewers to assess whether or not the participants received the exposure of interest.

**3. Was the exposure measured in a valid and reliable way?**

The study should clearly describe the method of measurement of exposure. Assessing validity requires that a 'gold standard' is available to which the measure can be compared. The validity of exposure measurement usually relates to whether a current measure is appropriate or whether a measure of past exposure is needed.

Reliability refers to the processes included in an epidemiological study to check repeatability of measurements of the exposures. These usually include intra-observer reliability and inter-observer reliability.

**4. Were confounding factors identified?**

Confounding has occurred where the estimated intervention exposure effect is biased by the presence of some difference between the comparison groups (apart from the exposure investigated/of interest). Typical confounders include baseline characteristics, prognostic factors, or concomitant exposures (e.g. smoking). A confounder is a difference between the comparison groups, and it influences the direction of the study results. A high quality study at the level of cohort design will identify the potential confounders and measure them (where possible). This is difficult for studies where behavioral, attitudinal or lifestyle factors may impact on the results.

**5. Were strategies to deal with confounding factors stated?**

Strategies to deal with effects of confounding factors may be dealt within the study design or in data analysis. By matching or stratifying sampling of participants, effects of confounding factors can be adjusted for. When dealing with adjustment in data analysis, assess the statistics used in the study. Most will be some form of multivariate regression analysis to account for the confounding factors measured. Look out for a description of statistical methods as regression methods such as logistic regression are usually employed to deal with confounding factors/variables of interest.

**6. Were the groups/participants free of the outcome at the start of the study (or at the moment of exposure)?**

The participants should be free of the outcomes of interest at the start of the study. Refer to the ‘methods’ section in the paper for this information, which is usually found in descriptions of participant/sample recruitment, definitions of variables, and/or inclusion/exclusion criteria.

**7. Were the outcomes measured in a valid and reliable way?**

Read the methods section of the paper. If for e.g. lung cancer is assessed based on existing definitions or diagnostic criteria, then the answer to this question is likely to be yes. If lung cancer is assessed using observer reported, or self-reported scales, the risk of over- or under-reporting is increased, and objectivity is compromised. Importantly, determine if the measurement tools used were validated instruments as this has a significant impact on outcome assessment validity. Having established the objectivity of the outcome measurement (e.g. lung cancer) instrument, it’s important to establish how the measurement was conducted. Were those involved in collecting data trained or educated in the use of the instrument/s? (e.g. radiographers). If there was more than one data collector, were they similar in terms of level of education, clinical or research experience, or level of responsibility in the piece of research being appraised?

**8. Was the follow up time reported and sufficient to be long enough for outcomes to occur?**

The appropriate length of time for follow up will vary with the nature and characteristics of the population of interest and/or the intervention, disease or exposure. To estimate an appropriate duration of follow up, read across multiple papers and take note of the range for duration of follow up. The opinions of experts in clinical practice or clinical research may also assist in determining an appropriate duration of follow up. For example, a longer timeframe may be needed to examine the association between occupational exposure to asbestos and the risk of lung cancer. It is important, particularly in cohort studies that follow up is long enough to enable the outcomes. However, it should be remembered that the research question and outcomes being examined would probably dictate the follow up time.

**9. Was follow up complete, and if not, were the reasons to loss to follow up described and explored?**

It is important in a cohort study that a greater percentage of people are followed up. As a general guideline, at least 80% of patients should be followed up. Generally a dropout rate of 5% or less is considered insignificant. A rate of 20% or greater is considered to significantly impact on the validity of the study. However, in observational studies conducted over a lengthy period of time a higher dropout rate is to be expected. A decision on whether to include or exclude a study because of a high dropout rate is a matter of judgement based on the reasons why people dropped out, and whether dropout rates were comparable in the exposed and unexposed groups. Reporting of efforts to follow up participants that dropped out may be regarded as an indicator of a well conducted study. Look for clear and justifiable description of why people were left out, excluded, dropped out etc. If there is no clear description or a statement in this regards, this will be a 'No'.

**10. Were strategies to address incomplete follow up utilized?**

Some people may withdraw due to change in employment, or some may die; however, it is important that their outcomes are assessed. Selection bias may occur as a result of incomplete follow up. Therefore, participants with unequal follow up periods must be taken into account in the analysis, which should be adjusted to allow for differences in length of follow up periods. This is usually done by calculating rates which use person-years at risk, i.e. considering time in the denominator.

**11. Was appropriate statistical analysis used?**

As with any consideration of statistical analysis, consideration should be given to whether there was a more appropriate alternate statistical method that could have been used. The methods section of cohort studies should be detailed enough for reviewers to identify which analytical techniques were used (in particular, regression or stratification) and how specific confounders were measured. For studies utilizing regression analysis, it is useful to identify if the study identified which variables were included and how they related to the outcome. If stratification was the analytical approach used, were the strata of analysis defined by the specified variables? Additionally, it is also important to assess the appropriateness of the analytical strategy in terms of the assumptions associated with the approach as differing methods of analysis are based on differing assumptions about the data and how it will respond.

*Moola S, Munn Z, Tufanaru C, Aromataris E, Sears K, Sfetcu R, Currie M, Qureshi R, Mattis P, Lisy K, Mu P-F. Chapter 7: Systematic reviews of etiology and risk . In: Aromataris E, Munn Z (Editors)*. JBI Manual for Evidence Synthesis. JBI, 2020. Available from <https://synthesismanual.jbi.global>

**Appendix D – Table S1. Risk of bias assessment of Cross-Sectional Studies**

| **Author, publication year** | **Q1** | **Q2** | **Q3** | **Q4** | **Q5** | **Q6** | **Q7** | **Q8** | **Overall appraisal** |
| --- | --- | --- | --- | --- | --- | --- | --- | --- | --- |
| Akinleye et al., 2020 (35) | Y | Y | Y | Y | N | N | N | Y | 5.0 |
| Alene et al., 2020 (36) | Y | Y | Y | Y | Y | Y | N | Y | 7.0 |
| Anyaka et al., 2024 (37) | Y | Y | Y | Y | N | N | N | Y | 5.0 |
| Aragaw et al., 2023 (38) | Y | Y | Y | Y | N | Y | N | Y | 6.0 |
| Azuogu et al., 2019 (39) | Y | Y | Y | Y | N | N | N | Y | 5.0 |
| Dairo et al., 2016 (40) | Y | Y | Y | Y | N | N | N | Y | 5.0 |
| Destaw et al., 2021 (41) | Y | Y | Y | Y | N | Y | N | Y | 6.0 |
| Dereje et al., 2021 (42) | Y | Y | Y | Y | N | Y | N | Y | 6.0 |
| DiAngi et al., 2011 (43) | U | Y | Y | U | N | N | N | Y | 4.0 |
| Enebe et al., 2021 (44) | Y | Y | Y | Y | N | N | N | Y | 5.0 |
| Ezenwa et al., 2013 (45) | Y | Y | Y | Y | N | N | U | Y | 5.5 |
| Humnesa et al., 2022 (46) | Y | Y | Y | Y | N | Y | N | Y | 6.0 |
| Kolek et al., 2022 (47) | Y | Y | Y | Y | Y | Y | N | Y | 7.0 |
| Lubeya et al., 2023 (48) | Y | Y | Y | Y | U | Y | N | Y | 6.5 |
| Mihretie et al., 2022 (49) | Y | Y | Y | Y | N | Y | N | Y | 6.0 |
| Milondzo et al., 2021 (50) | Y | Y | Y | Y | N | N | N | Y | 5.0 |
| Morhason-Bello et al., 2015 (51) | Y | Y | Y | U | N | Y | N | Y | 5.5 |
| Ndejjo et al., 2017 (52) | Y | Y | Y | U | N | Y | N | Y | 5.5 |
| Okunade et al., 2017 (54) | Y | Y | U | Y | N | N | N | Y | 4.5 |
| Okunowo et al., 2021 (25) | Y | Y | N | Y | N | Y | N | Y | 5.0 |
| Rabiu et al., 2020 (26) | U | U | Y | U | N | N | N | Y | 3.5 |
| Sinshaw et al., 2022 (55) | Y | Y | Y | Y | Y | Y | N | Y | 7.0 |
| Tsige et al., 2024 (36) | Y | Y | Y | Y | N | Y | N | Y | 6.0 |
| Zibako et al., 2021 (58) | Y | Y | Y | Y | N | N | U | Y | 5.5 |

*Y: Yes; N: No; U: Unclear; NA: Not Applicable; Low quality: 0-3.9; Moderate quality: 4.0-6.9; High quality: 7.0-8.0*

*Q1: Were the criteria for inclusion in the sample clearly defined?; Q2: Were the study subjects and the setting described in detail?; Q3: Was the exposure measured in a valid and reliable way?; Q4: Were objective, standard criteria or definitions used for identifying appropriate study participants based on their status as parents/guardians/caregivers?; Q5: Were confounding factors identified?; Q6: Were strategies to deal with confounding factors stated?; Q7: Were the outcomes measured in a valid and reliable way?; Q8: Was appropriate statistical analysis used?*

**Appendix E –** **Table S2.** **Risk of bias assessment of Cohort Studies**

| **Author, publication year** | **Q1** | **Q2** | **Q3** | **Q4** | **Q5** | **Q6** | **Q7** | **Q8** | **Q9** | **Q10** | **Q11** | **Overall appraisal** |
| --- | --- | --- | --- | --- | --- | --- | --- | --- | --- | --- | --- | --- |
| Nkwonta et al., 2021 (53) | Y | Y | Y | N | Y | NA | Y | Y | N | N | Y | 7.0 |
| Vermandere et al., 2014 (57) | Y | Y | Y | N | Y | NA | N | Y | Y | Y | Y | 8.0 |

*Y: Yes; N: No; U: Unclear; NA: Not Applicable; Low quality: 0-4.9 points; Moderate quality: 5.0-8.9 points; High Quality: 9.0-11.0 points*

*Q1: Were the same participants recruited both before and after the intervention?; Q2: Were the exposures measured in a similar way before and after the intervention?; Q3: Was the exposure measured in a valid and reliable way?; Q4: Were confounding factors identified?; Q5: Were strategies to deal with confounding factors stated?; Q6: Were the groups/participants free of the outcome at the start of the study (or at the time of exposure)?; Q7: Were the outcomes measured in a valid and reliable way?; Q8: Was the follow up time reported and sufficient to be long enough for outcomes to occur?; Q9: Was follow up complete, and if not, were the reasons to loss to follow up described and explored?; Q10: Were strategies to address incomplete follow up utilized?; Q11: Was appropriate statistical analysis used?*

**Appendix F – Table S3. Association between residence and parents’ awareness of HPV vaccination**

| **Author, publication year** | **Residence** | **N (%)** | **OR [95% CI]** | **AOR [95% CI]** |
| --- | --- | --- | --- | --- |
| Humnesa et al., 2022 (46) | Rural  Urban | 185 (35.1)  64 (69.6) | Ref  4.23 (2.62 – 6.82) | Ref  1.76 (0.47 – 6.57) |
| Zibako et al.,  2021 (58) | Urban  Rural | * | Ref  1.31 (0.79 – 2.19) | *** |

** Information on N (%) was missing*

**** Information on AOR was missing*

**Appendix G – Table S4. Association between occupation and parents’ awareness of HPV vaccination**

| **Author, year** | **Occupation** | **N (%)** | **OR [95% CI]** | **AOR [95% CI]** |
| --- | --- | --- | --- | --- |
| Mihretie, 2022 | Housewife  Government employee  Self-employees  Merchants | 218 (16.4)  60 (48.8)  31 (33.7)  69 (37.7) | 1  4.86 (2.62 – 9.01)  2.59 (1.33 – 5.05)  3.09 (1.72 – 5.56) | 1  5.46 (2.42-9.34)  2.57 (1.25-5.27)  3.10 (1.62-5.93) |
| Morhason-Bello, 2015 | None  Unskilled  Semi-skilled  Skilled | 9 (3.7)  4 (3.8)  18 (3.3)  14 (19.2) | Ref  1.04 (0.31 – 3.46)  0.89 (0.40 – 2.02)  6.25 (2.58 – 15.13) | *** |
| Sinshaw, 2022 | Civil servant  Self-employed  Merchant  Farmer  Housewife  Others | 139 (75.9)  5 (35.7)  61 (39.9)  2 (25.0)  74 (32.6)  5 (31.3) | Ref  0.18 (0.06 – 0.55)  0.21 (0.13 – 0.34)  0.11 (0.21 – 0.54)  0.15 (0.10 – 0.24)  0.14 (0.05 – 0.44) | Ref  0.34 (0.04 – 2.60)  1.60 (0.50 – 5.12)  6.27 (0.61 – 64.86)  1.06 (0.30 – 3.73)  0.56 (0.08 – 3.82) |
| Tsige et al., 2024 | Governmental  Self-employed  Housewife  Others | * | Ref  0.18 (0.06 – 0.55)  0.21 (0.13 – 0.34)  0.11 (0.21 – 0.54) | Ref  0.34 (0.043 – 2.60)  1.60 (0.50 – 5.12)  6.27 (0.61 – 64.86) |
| Zibako, 2021 | Unemployed  Employed | * | Ref  0.73 (0.31 – 1.72) | *** |

** Information on N (%) was missing*

**** Information on AOR was missing*

**Appendix H – Table S5. Association between income and parents’ willingness to vaccinate daughters**

| **Author, publication year** | **Income** | **N (%)** | **OR [95% CI]** | **AOR [95% CI]** |
| --- | --- | --- | --- | --- |
| Alene et al., 2020 (36) | Poor  Medium  Rich | 203 (69.28)  254 (83.28)  274 (91.03) | Ref  2.20 (1.49 – 3.26)  4.49 (2.82 – 7.17) | Ref  2.04 (1.27 – 3.27)  3.44 (1.97 – 6.01) |
| Aragaw et al., 2023 (38) | < Br 2000  Br 2000 – 4000  > Br 4000 | 21 (80.8)  180 (71.7)  369 (83.1) | Ref  0.60 (0.22 – 1.66)  1-17 (0.43 – 3.20) | *** |
| Dairo et al., 2016 (40) | < ₦20 000  ₦20–50 000  > ₦50 000 | 168 (70.0)  241 (82.0)  71 (91.0) | Ref  4.3 (1.9 – 9.9)  1.9 (1.3 – 2.9) | *** |
| Destaw et al., 2021 (41) | <42$  42$-85$  >85$ | 108 (81.8)  101 (72.1)  190 (82.6) | 0.90 (0.54 – 1.65)  0.50 (0.33 – 0.90)  Ref | 1.60 (0.88 – 3.02)  0.70 (0.40 – 1.19)  Ref |
| Dereje et al., 2021 (42) | ≤100$  >100$ | 148 (37.2)  250 (62.8) | Ref  2.82 (1.20 – 6.59) | Ref  2.48 (1.08 – 6.34) |
| DiAngi et al., 2011 (43) | No regular income  <$360  >$360 | 157 (89.0)  100 (92.0)  69 (82.0) | Ref  1.42 (0.62 – 3.23)  0.59 (0.28 – 1.21) | *** |
| Lubeya et al., 2023(48) | Poorest/poorer/middle  Richer/richest | 121 (49.4)  94 (60.6) | Ref  1.23 (0.88 – 1.72) | *** |
| Ndejjo et al., 2017 (52) | Less than $40  $40 and above | 552 (91.8)  261 (96.3) | Ref  0.95 (0.78 –1.7) | *** |
| Sinshaw et al., 2022 (55) (M) | < Br 600  Br 601 – 1650  Br 1651 – 3200  Br 3201 – 5250  Br 5251 – 7800  Br 7801 – 10 900  > Br 10 900 | 66 (70.2)  108 (75.0)  122 (78.2)  76 (83.5)  58 (75.7)  29 (80.6)  6 (100.0) | Ref  1.27 (0.71 – 2.28)  1.52 (0.85 – 2.73)  2.15 (1.06 – 4.37)  1.54 (0.76 – 3.12)  1.76 (0.69 – 4.48)  - | Ref  0.81 (0.40 – 1.66)  1.57 (0.68 – 3.61)  1.67 (0.47 – 5.91)  1.08 (0.27 – 4.36)  0.67 (0.14 – 3.28)  - |
| Sinshaw et al., 2022  (55) (F) | < Br 600  Br 601 – 1650  Br 1651 – 3200  Br 3201 – 5250  Br 5251 – 7800  Br 7801 – 10 900  > Br 10 900 | 6 (75.0)  7 (53.8)  63 (75.0)  101 (72.1)  90 (79.6)  88 (83.8)  11 (78.6) | Ref  0.39 (0.06 – 2.70)  1.00 (0.19 – 5.34)  0.86 (0.17 – 4.46)  1.30 (0.25 – 6.89)  1.73 (0.32 – 9.28)  1.22 (0.16 – 9.47) | *** |

*(M) indicates mothers, (F) indicates fathers.*

₦ = Nigerian Naira, Br = Ethiopian Birr

** Information on N (%) was missing; ** Information on OR was missing; *** Information on AOR was missing*

**Appendix I – Table S6: Association between residence and parents’ willingness to vaccinate daughters**

| **Author, publication year** | **Residence** | **N (%)** | **OR [95% CI]** | **AOR [95% CI]** |
| --- | --- | --- | --- | --- |
| Alene et al., 2020 (36) | Rural  Urban | 215 (83.3)  516 (80.5) | Ref  1.21 (0.82 – 1-77) | Ref  1.34 (0.76 – 2.39) |
| Destaw et al., 2021 (41) | Rural  Urban | 211 (76.4)  188 (83.2) | Ref  1.50 (0.97 – 2.38) | Ref  1.50 (0.96 – 2-49) |
| DiAngi et al., 2011 (43) | Rural  Urban | 119 (93.0)  202 (85.0) | 2.29 (1.06 – 4.93)  Ref |  |
| Ndejjo et al., 2017 (52) | Rural  Semiurban/urban | 542 (91.9)  271 (96.1) | Ref  2.18 (1.12 – 4.27) |  |
| Vermandere et al., 2014  (57) | Rural  Urban | 96 (85.0)  155 (90.6) | Ref  1.72 (0.83 – 3.55) |  |

*** Information on AOR was missing in some articles.*

**Appendix J – Table S7: Association between occupation and parents’ acceptability of HPV vaccination**

| **Author, year** | **Occupation** | **N (%)** | **OR [95% CI]** | **AOR [95% CI]** |
| --- | --- | --- | --- | --- |
| Aragaw et al., 2023 | Housewife  Merchant  Government employee  Self-employee  Others | 149 (73.8)  138 (75.0)  225 (90.4)  55 (69.6)  3 (42.9) | Ref  1.07 (0.68 – 1.69)  3.34 (1.97 – 5.64)  0.82 (0.46 – 1.45)  0.27 (0.06 – 1.23) | Ref  0.87 (0.49 – 1.53)  1.61 (0.66 – 3.95)  0.84 (0.4 – 1.73)  1.88 (0.29 – 12.11) |
| Dairo, 2016 | Others  Businessman/woman  Artisan  Public servant  Civil servant | 51 (60.0)  153 (74.6)  64 (66.7)  148 (93.7)  63 (94.0) | Ref  2.0 (1.1 – 3.3)  1.3 (0.7 – 2.4)  9.9 (4.6 – 21.4)  1.0 (3.5 – 31.5) |  |
| Kolek, 2022 | Other  Self-employed  Formal employment |  | 0.92 (0.48 – 1.76) |  |
| Lubeya et al., 2023 | Employed  Unemployed | 170 (51.5)  45 (64.3) | Ref  1.25 (0.82 – 1.89) |  |
| Mihretie, 2022 | Government employees  Others* | 64 (28.2)  114 (27.7) | Ref  1.02 (0.71 – 1.469) | Ref  1.02 (0.51 – 2.01) |
| Milondzo, 2021 | Unemployed  Part-time employed  Self-employed  Employed | 4 (8.0)  10 (16.4)  13 (10.6)  47 (14.9) | Ref  1.71 (0.48 – 6.04)  1.11 (0.33 – 3.71)  1.70 (0.56 – 5.10) |  |
| Ndejjo, 2017 | Farming  Others | 470 (95.5)  343 (90.3) | Ref  0.94 (0.91 – 0.98) |  |
| Okunowo, 2021 | Unemployed  Employed |  | Ref  2.79 (1.22 – 6.42) | Ref  4.78 (1.78 – 12.86) |
| Rabiu, 2020  (M) | Unskilled  Semi-skilled  Skilled | 86 (58.1)  47 (68.1)  96 (95.0) | 0.65 (0.36 – 1.19)  Ref  8.99 (3.20 – 25.22) |  |
| Rabiu, 2020  (F) | Unskilled  Semi-skilled  Skilled | 47 (73.4)  118 (68.6)  57 (76.0) | 1.27 (0.67 – 2.40)  Ref  1.45 (0.78 – 2.70) |  |
| Sinshaw, 2022 (M) | Civil servant  Self-employed  Merchant  Farmer  Housewife  Others | 151 (82.5)  8 (57.1)  109 (71.2)  7 (87.5)  177 (78.0)  13 (81.3) | Ref  0.28 (0.09 – 0.87)  0.53 (0.31 – 0.88)  1.48 (0.18 – 12.48)  0.75 (0.46 – 1.23)  0.92 (0.25 – 3.41) | Ref  0.67 (0.11 – 3.96)  1.72 (0.45 – 6.49)  12.25 (0.87 – 171.92)  2.69 (0.65 – 11.21)  2.67 (0.30 – 23.36) |
| Sinshaw, 2022 (F) | Civil servant  Self-employed  Merchant  Farmer  Others | 179 (83.6)  38 (67.9)  124 (72.5)  2 (66.7)  23 (69.7) | Ref  0.41 (0.21 – 0.81)  0.51 (0.32 – 0.85)  0.39 (0.04 – 4.43)  0.45 (0.20 – 1.03) | Ref  0.57 (0.22 – 1.48)  0.58 (0.26 – 1.30)  0.60 (0.04 – 9.52)  0.49 (0.16 – 1.56) |

*Self-employees, farmer, merchant, daily labor

**Appendix K – GRADE Assessment - Funnel plots**


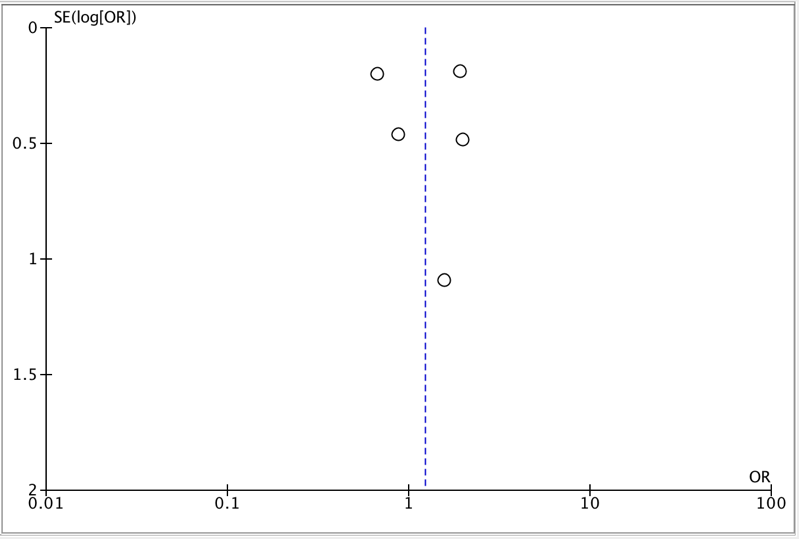

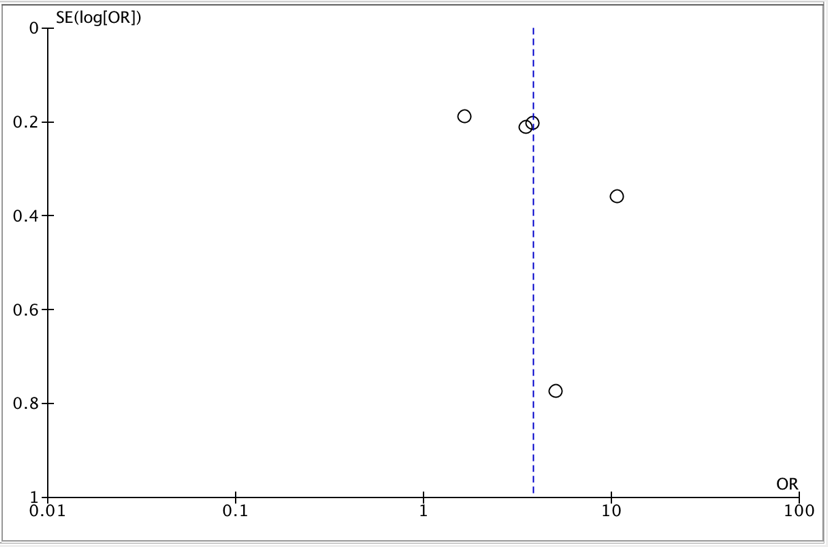


Figure 2: Funnel plot of association between mod/low income and awareness

Figure 1: Funnel plot of association between education and awareness


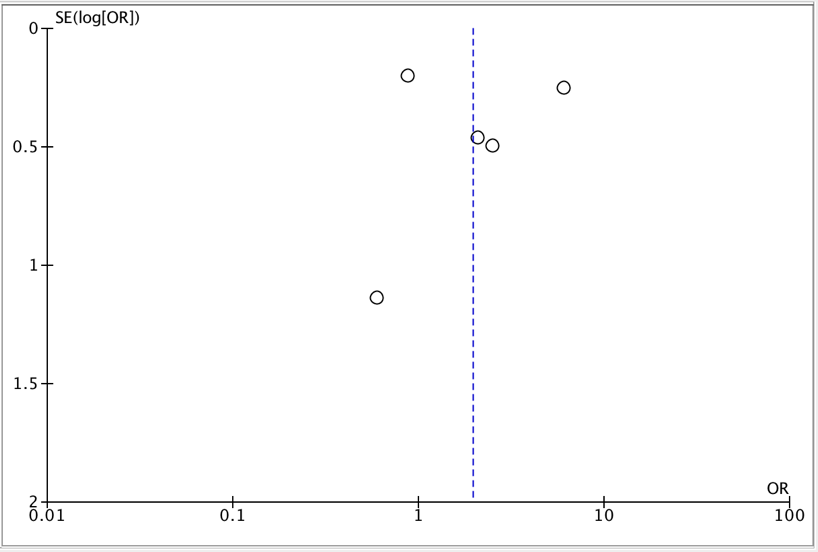

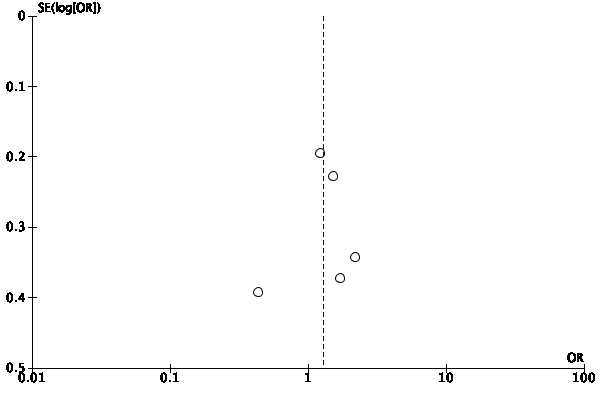


Figure 4: Funnel plot of association between residence and awareness

Figure 3: Funnel plot of association between high/low income and awareness


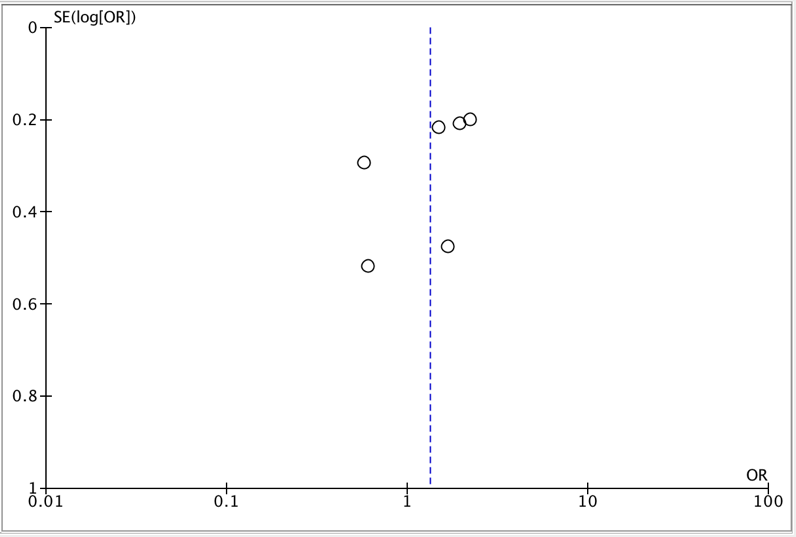

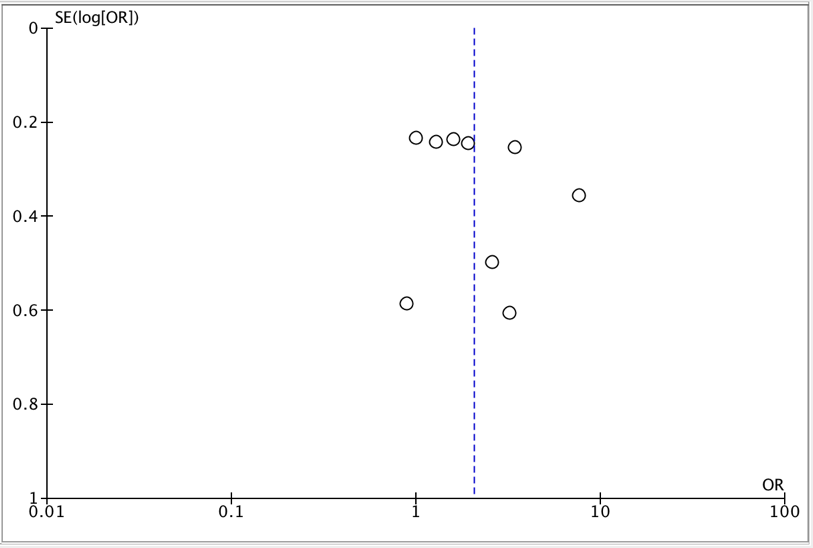


Figure 5: Funnel plot of association between education and acceptability

Figure 6: Funnel plot of association between mod/low income and acceptability


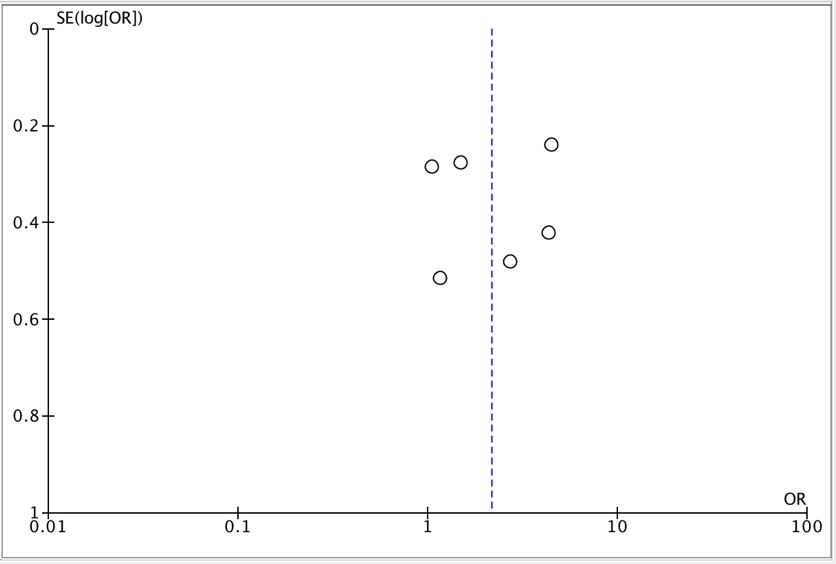


Figure 7: Funnel plot of association between high/low income and acceptability

## Appendix L – GRADE Assessment - Explanations

EXPLANATIONS

Association between education and acceptability of HPV vaccination

As the overall risk of bias was not serious, the evidence was not downgraded. However, there were some concerns as five out of seven studies were of moderate quality. As there was considerable heterogeneity (I^2^ = 77%), inconsistency was downgraded by one level. The precision was not downgraded as the confidence intervals of the pooled odds ratio were narrow (2.01 [1.35 – 3.15]). Indirectness was downgraded by one level since the analysis only included studies from two countries, Nigeria and Ethiopia, out of 46 countries in SSA, which may limit the generalizability of the findings to the entire region. The observed symmetry in the funnel plot suggests that publication bias was unlikely (Fig.1 in Appendix F).

Association between education and awareness of HPV vaccination

As the overall risk of bias was not serious, the evidence was not downgraded. There were some concerns as four out of five studies were of moderate quality, while only one was of high quality. There was downgrading by one level for inconsistency, imprecision and indirectness, due to substantial heterogeneity (I^2^ = 84%), wide confidence intervals (3.81 [2.11 – 6.88]) and inclusion of only two countries in SSA, Nigeria and Ethiopia. The indication of publication bias in the funnel plot (Fig.2 in Appendix F) led to downgrading by one level.

Association between income and acceptability of HPV (high income vs. low income)

As the overall risk of bias was not serious, the evidence was not downgraded. There were some concerns as three out of five studies were of moderate quality. As there was considerable heterogeneity (I^2^ = 77%), inconsistency was downgraded by one level. The precision was not downgraded as the confidence intervals of the pooled odds ratio were narrow (2.16 [1.21 – 3.84]). Indirectness was downgraded by one level since the analysis only included studies from two countries in SSA, Nigeria and Ethiopia. The observed symmetry in the funnel plot suggests that publication bias was unlikely (Fig.3 in Appendix F).

Association between income and acceptability of HPV (moderate income vs. low income)

As the overall risk of bias was not serious, the evidence was not downgraded. There were some concerns as three out of five studies were of moderate quality. As there was considerable heterogeneity (I^2^ = 74%), inconsistency was downgraded by one level. The precision was not downgraded as the confidence intervals of the pooled odds ratio were narrow (1.33 [0.86 – 2.07]). Indirectness was downgraded by one level since the analysis only included studies from two countries in SSA, Nigeria and Ethiopia. The observed symmetry in the funnel plot suggests that publication bias was unlikely (Fig.4 in Appendix F).

Association between income and awareness of HPV (high income vs. low income)

As the overall risk of bias was not serious, the evidence was not downgraded. There were some concerns as three out of four studies were of moderate quality. There was downgrading by one level for inconsistency, imprecision and indirectness, due to high heterogeneity (I^2^ = 89%), wide confidence intervals (1.96 [0.72 – 5.29]) and inclusion of only two countries in SSA, Nigeria and Ethiopia. The observed symmetry in the funnel plot suggests that publication bias was unlikely (Fig.5 in Appendix F).

Association between income and awareness of HPV (moderate income vs. low income)

As the overall risk of bias was not serious, the evidence was not downgraded. There were some concerns as three out of four studies were of moderate quality. As there was considerable heterogeneity (I^2^ = 74 %), inconsistency was downgraded by one level. Imprecision was not downgraded as the confidence interval was narrow (1.22 [0.67 - 2.23]). Indirectness was downgraded by one level since the analysis only included studies from two countries in SSA, Nigeria and Ethiopia. The observed symmetry in the funnel plot suggests that publication bias was unlikely (Fig.6 in Appendix F).

Association between residence and acceptability

As the overall risk of bias was not serious a downgrade was not necessary, with two high-quality studies, one moderate-quality and one low-quality study. For inconsistency the evidence was downgraded by one level as a substantial heterogeneity was observed (I^2^ = 73 %). The precision was not downgraded as the confidence intervals were narrow (1.01 [0.60 – 1.70]). Indirectness was downgraded by one level as the analysis only included three out of 46 countries in SSA (Kenya, Botswana, and Ethiopia). The observed symmetry in the funnel plot suggests that publication bias was unlikely (Fig. 7 in Appendix F).

## Appendix M – PRISMA 2020 Checklist

| **Section and Topic** | **Item #** | **Checklist item** | **Location where item is reported** |
| --- | --- | --- | --- |
| **TITLE** | | |  |
| Title | 1 | Identify the report as a systematic review. | Page 1 |
| **ABSTRACT** | | |  |
| Abstract | 2 | See the PRISMA 2020 for Abstracts checklist. | Page 2 |
| **INTRODUCTION** | | |  |
| Rationale | 3 | Describe the rationale for the review in the context of existing knowledge. | Page 4 – 6 |
| Objectives | 4 | Provide an explicit statement of the objective(s) or question(s) the review addresses. | Page 6 |
| **METHODS** | | |  |
| Eligibility criteria | 5 | Specify the inclusion and exclusion criteria for the review and how studies were grouped for the syntheses. | Page 6 – 7 |
| Information sources | 6 | Specify all databases, registers, websites, organisations, reference lists and other sources searched or consulted to identify studies. Specify the date when each source was last searched or consulted. | Page 7 |
| Search strategy | 7 | Present the full search strategies for all databases, registers and websites, including any filters and limits used. | Appendix A |
| Selection process | 8 | Specify the methods used to decide whether a study met the inclusion criteria of the review, including how many reviewers screened each record and each report retrieved, whether they worked independently, and if applicable, details of automation tools used in the process. | Page 7 – 9 |
| Data collection process | 9 | Specify the methods used to collect data from reports, including how many reviewers collected data from each report, whether they worked independently, any processes for obtaining or confirming data from study investigators, and if applicable, details of automation tools used in the process. | Page 8 – 9 |
| Data items | 10a | List and define all outcomes for which data were sought. Specify whether all results that were compatible with each outcome domain in each study were sought (e.g. for all measures, time points, analyses), and if not, the methods used to decide which results to collect. | Page 8 – 9 |
|  | 10b | List and define all other variables for which data were sought (e.g. participant and intervention characteristics, funding sources). Describe any assumptions made about any missing or unclear information. | Page 8 – 9 |
| Study risk of bias assessment | 11 | Specify the methods used to assess risk of bias in the included studies, including details of the tool(s) used, how many reviewers assessed each study and whether they worked independently, and if applicable, details of automation tools used in the process. | Page 8 – 9 |
| Effect measures | 12 | Specify for each outcome the effect measure(s) (e.g. risk ratio, mean difference) used in the synthesis or presentation of results. | Page 8 – 9 |
| Synthesis methods | 13a | Describe the processes used to decide which studies were eligible for each synthesis (e.g. tabulating the study intervention characteristics and comparing against the planned groups for each synthesis (item #5)). | Page 9 – 10 |
|  | 13b | Describe any methods required to prepare the data for presentation or synthesis, such as handling of missing summary statistics, or data conversions. | Page 7 – 9 |
|  | 13c | Describe any methods used to tabulate or visually display results of individual studies and syntheses. | Page 9 |
|  | 13d | Describe any methods used to synthesize results and provide a rationale for the choice(s). If meta-analysis was performed, describe the model(s), method(s) to identify the presence and extent of statistical heterogeneity, and software package(s) used. | Page 9 |
|  | 13e | Describe any methods used to explore possible causes of heterogeneity among study results (e.g. subgroup analysis, meta-regression). | Page 9 – 10 |
|  | 13f | Describe any sensitivity analyses conducted to assess robustness of the synthesized results. |  |
| Reporting bias assessment | 14 | Describe any methods used to assess risk of bias due to missing results in a synthesis (arising from reporting biases). | Page 8 – 9 |
| Certainty assessment | 15 | Describe any methods used to assess certainty (or confidence) in the body of evidence for an outcome. | Page 9 – 10 |
| **RESULTS** | | |  |
| Study selection | 16a | Describe the results of the search and selection process, from the number of records identified in the search to the number of studies included in the review, ideally using a flow diagram. | Page 11 |
|  | 16b | Cite studies that might appear to meet the inclusion criteria, but which were excluded, and explain why they were excluded. | Page 11 |
| Study characteristics | 17 | Cite each included study and present its characteristics. | Page 12 – 15 |
| Risk of bias in studies | 18 | Present assessments of risk of bias for each included study. | Appendix D & E |
| Results of individual studies | 19 | For all outcomes, present, for each study: (a) summary statistics for each group (where appropriate) and (b) an effect estimate and its precision (e.g. confidence/credible interval), ideally using structured tables or plots. | Page 18 – 26 |
| Results of syntheses | 20a | For each synthesis, briefly summarise the characteristics and risk of bias among contributing studies. | Page 18 – 26 |
|  | 20b | Present results of all statistical syntheses conducted. If meta-analysis was done, present for each the summary estimate and its precision (e.g. confidence/credible interval) and measures of statistical heterogeneity. If comparing groups, describe the direction of the effect. | Page 18 – 26  Appendix F – J |
|  | 20c | Present results of all investigations of possible causes of heterogeneity among study results. | Page 18 – 26 |
|  | 20d | Present results of all sensitivity analyses conducted to assess the robustness of the synthesized results. |  |
| Reporting biases | 21 | Present assessments of risk of bias due to missing results (arising from reporting biases) for each synthesis assessed. | Page 18 – 26 |
| Certainty of evidence | 22 | Present assessments of certainty (or confidence) in the body of evidence for each outcome assessed. | Page 27 |
| **DISCUSSION** | | |  |
| Discussion | 23a | Provide a general interpretation of the results in the context of other evidence. | Page 28 – 30 |
|  | 23b | Discuss any limitations of the evidence included in the review. | Page 30 |
|  | 23c | Discuss any limitations of the review processes used. | Page 30 |
|  | 23d | Discuss implications of the results for practice, policy, and future research. | Page 31 – 32 |
| **OTHER INFORMATION** | | |  |
| Registration and protocol | 24a | Provide registration information for the review, including register name and registration number, or state that the review was not registered. |  |
|  | 24b | Indicate where the review protocol can be accessed, or state that a protocol was not prepared. | Page 6 |
|  | 24c | Describe and explain any amendments to information provided at registration or in the protocol. |  |
| Support | 25 | Describe sources of financial or non-financial support for the review, and the role of the funders or sponsors in the review. |  |
| Competing interests | 26 | Declare any competing interests of review authors. |  |
| Availability of data, code and other materials | 27 | Report which of the following are publicly available and where they can be found: template data collection forms; data extracted from included studies; data used for all analyses; analytic code; any other materials used in the review. |  |

Page MJ, McKenzie JE, Bossuyt PM, Boutron I, Hoffmann TC, Mulrow CD, et al. The PRISMA 2020 statement: an updated guideline for reporting systematic reviews. BMJ 2021;372:n71. doi: 10.1136/bmj.n71
